# Nanocatalytic Neuroprotection and Neurological Recovery Post‐Traumatic Brain Injury

**DOI:** 10.1002/advs.202505962

**Published:** 2025-08-11

**Authors:** Xinjie Hong, Liang Zhao, Xianzheng Sang, Chao Ma, Meiqi Chang, Xinran Song, Wei Feng, Tao Xu, Li Ding, Yu Chen, Lijun Hou

**Affiliations:** ^1^ Department of Neurosurgery The Second Affiliated Hospital of Naval Medical University Shanghai 200003 P. R. China; ^2^ Laboratory Center, Shanghai Municipal Hospital of Traditional Chinese Medicine Shanghai University of Traditional Chinese Medicine Shanghai 200071 P. R. China; ^3^ Department of Medical Ultrasound, Shanghai Tenth People's Hospital, Tongji University Cancer Center, School of Medicine Tongji University Shanghai 200072 P. R. China; ^4^ Materdicine Lab School of Life Sciences Shanghai University Shanghai 200444 P. R. China

**Keywords:** nanocatalytic therapy, nanoenzyme, neuroprotection, traumatic brain injury

## Abstract

Traumatic brain injury (TBI) is a severe neurological disorder requiring novel therapeutic strategies. We developed ultrasmall catalytic Ce_0.7_Zr_0.3_O_2_ nanozymes (CZs) and investigated their neuroprotective potential in combination with nimodipine, a calcium channel blocker. CZs effectively alleviated oxidative stress but were insufficient against calcium‐mediated neuronal injury, while nimodipine alone inadequately mitigated oxidative damage. Combined therapy preserved blood–brain barrier integrity, reduced oxidative stress and neuroinflammation, and inhibited neuronal apoptosis, with CZs exerting potent effects even at low doses. Nimodipine synergistically regulated calcium overload, suppressed CaMKII activation, and enhanced functional recovery during later TBI stages. Notably, nimodipine promoted preferential accumulation of CZs in injured brain tissue, further amplifying neuroprotection. Behavioral and histological analyses confirmed significant improvements in cognitive and motor outcomes, indicating superior efficacy of the combined treatment over either agent alone. These findings highlight a promising strategy integrating nanozyme‐based antioxidative therapy with calcium channel blockade for comprehensive TBI management, offering translational potential for clinical application.

## Graphical abstract

This study demonstrates that combining Ce_0.7_Zr_0.3_O_2_ nanozymes with nimodipine enhances neuroprotection after traumatic brain injury. The therapy reduces oxidative stress, neuronal apoptosis, and improves blood–brain barrier integrity, with nanozymes effective at low doses. Nimodipine promotes targeted brain accumulation of nanozymes, highlighting a promising strategy integrating nanomaterials and calcium channel blockade for comprehensive TBI management.

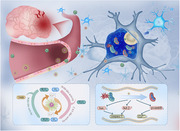

## 1 Introduction

Traumatic Brain Injury (TBI) is a prevalent and critical medical affliction of the central nervous system that is commonly observed in military conflicts and accidents.^[^ ^1^, ^2^ ^]^ TBI exhibits a substantial incidence of disability and mortality, making it a critical condition that disrupts neurological function.^[^ ^3^ ^]^ In accordance with the pathophysiology underlying TBI, it encompasses a primary mechanical insult followed by a subsequent cascade of cellular and functional impairments.^[^ ^4^, ^5^ ^]^ Notably, these secondary injuries are characterized by neuronal dysfunction or potential demise triggered by oxidative stress,^[^ ^6^ ^]^ calcium overload,^[^ ^7^ ^]^ mitochondrial dysfunction,^[^ ^8^, ^9^ ^]^ myelin sheath damage,^[^ ^10^ ^]^ in addition to the aberrant activation of microglia^[^ ^11^ ^]^ and astrocytes,^[^ ^12^ ^]^ culminating in a supplementary impact on overall functional recovery. Calcium overload has been identified as a pivotal contributor to the progression of secondary damage following TBI.^[^ ^13^, ^14^ ^]^ The dysregulation of intracellular calcium impels neuronal oxidative stress and fosters excessive production of reactive oxygen species (ROS), thereby eliciting a cascade of detrimental events culminating in neuronal cell death.^[^ ^15^, ^16^ ^]^

In recent years, there has been significant recognition and widespread implementation of biologically active nanozymes in diverse biomedical applications due to their distinctive enzymatic properties.^[^ ^17^ ^]^ Cerium‐based nanoparticles, in particular, have garnered escalating interest in the realm of nanozymes due to their exceptional enzyme‐like catalytic activities, which encompass superoxide dismutase (SOD), catalase (CAT), peroxidase (POD), and sustained efficacy in scavenging ROS.^[^ ^18^, ^19^, ^20^ ^]^ Capitalizing on these attributes, our research team has successfully synthesized potent therapeutic agents in the form of ceria–zirconia nanoparticles (CZs). These nanoparticles showcase remarkable potential in mitigating TBI and exhibiting superior efficacy in addressing central nervous system (CNS) disorders.^[^ ^21^, ^22^ ^]^ Notably, the reversible redox transitions between Ce^3+^ and Ce^4+^ within the ceria–zirconia nanoparticles framework facilitate the creation of oxygen vacancies and low valence states, critically contributing to the eradication of ROS generated by oxidative stress and subsequent attenuation of inflammation.^[^ ^23^, ^24^ ^]^ Significantly, the incorporation of an optimal percentage of zirconium into the nanoparticle composition effectively maintains a high Ce^3+^ content within the system. This strategic approach further enhances the therapeutic capabilities of the ceria–zirconia nanoparticles.^[^ ^25^, ^26^ ^]^

Concurrently, the blocker of calcium channels has the potential to inhibit calcium overload and consequently mitigate neuronal cell death when combined with the enzyme activity of cerium zirconium oxide.^[^ ^27^, ^28^ ^]^ Notably, nimodipine,^[^ ^29^ ^]^ which is recognized as a potent neuroprotective agent,^[^ ^30^, ^31^ ^]^ can be effectively paired with nano‐enzymes, thus demonstrating superior neurological enhancement. The effective traversal of nanoparticles across the blood‐brain barrier (BBB) is also imperative for the treatment of CNS disorders.^[^ ^32^, ^33^, ^34^ ^]^ To surmount this obstacle, we intend to harness the synergistic effects of co‐administration to ensure a more impactful role for the nanoparticles. Our objective is to combine nimodipine as a synergistic delivery agent with cerium zirconium oxide nano‐enzymes in order to augment their accumulation and efficacy in the brain, particularly in regions severely impacted by TBI. This integrated approach is anticipated to surmount the challenges stemming from the limited concentration of nanoparticles in the brain and furnish an effective therapeutic strategy for CNS disorders.

**Scheme 1:**
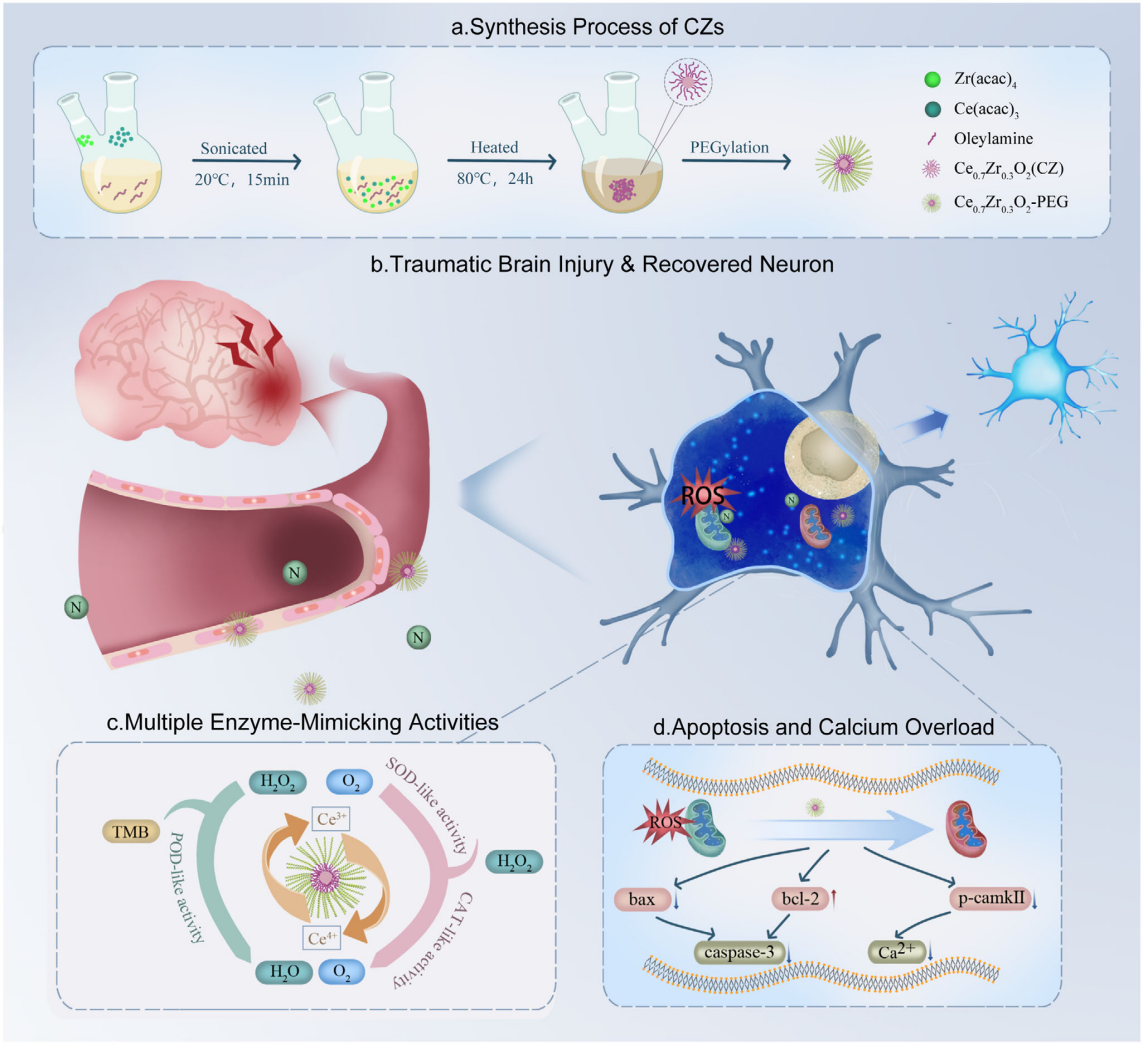
Synthesis, mechanistic actions, and neuroprotective effects of Ce_0.7_Zr_0.3_O_2_ nanozymes (CZs) in traumatic brain injury (TBI). a) Schematic illustration of the synthesis process of CZs. b) Mechanistic depiction of the significant alleviation of neuronal cell death following TBI by CZs. c) Mechanistic illustration of CZs mimics the activities of multiple biological enzymes. d) Mechanistic depiction of the alleviation of apoptosis and calcium overload in neuronal cells by CZs.

This study presents a novel therapeutic paradigm that leverages the functionality of nimodipine as both a calcium antagonist and an enhancer of the cerebral accumulation of ultrasmall ceria‐zirconia nanozymes (CZs). The synergistic combination of CZs and nimodipine was systematically evaluated in a murine model of TBI (**Scheme** **1**). CZs exhibited robust ROS‐scavenging activity at low concentrations, while the co‐treatment outperformed monotherapies in mitigating oxidative stress, suppressing neuronal apoptosis, and restoring neurological function. Notably, the use of minimal doses effectively minimized neurotoxicity. These findings highlight a clinically translatable, low‐toxicity, dual‐targeted strategy that integrates nanocatalytic and pharmacological modalities for advanced neuroprotection in TBI.

## 2 Results and Discussion

### 2.1 Preparation, Characterization of Ceria‐Zirconia Nanozymes (CZ) and Phospholipid‐Polyethylene Glycol (PEG)‐Modified CZ Nanozymes (CZs)

**Figure 1:**
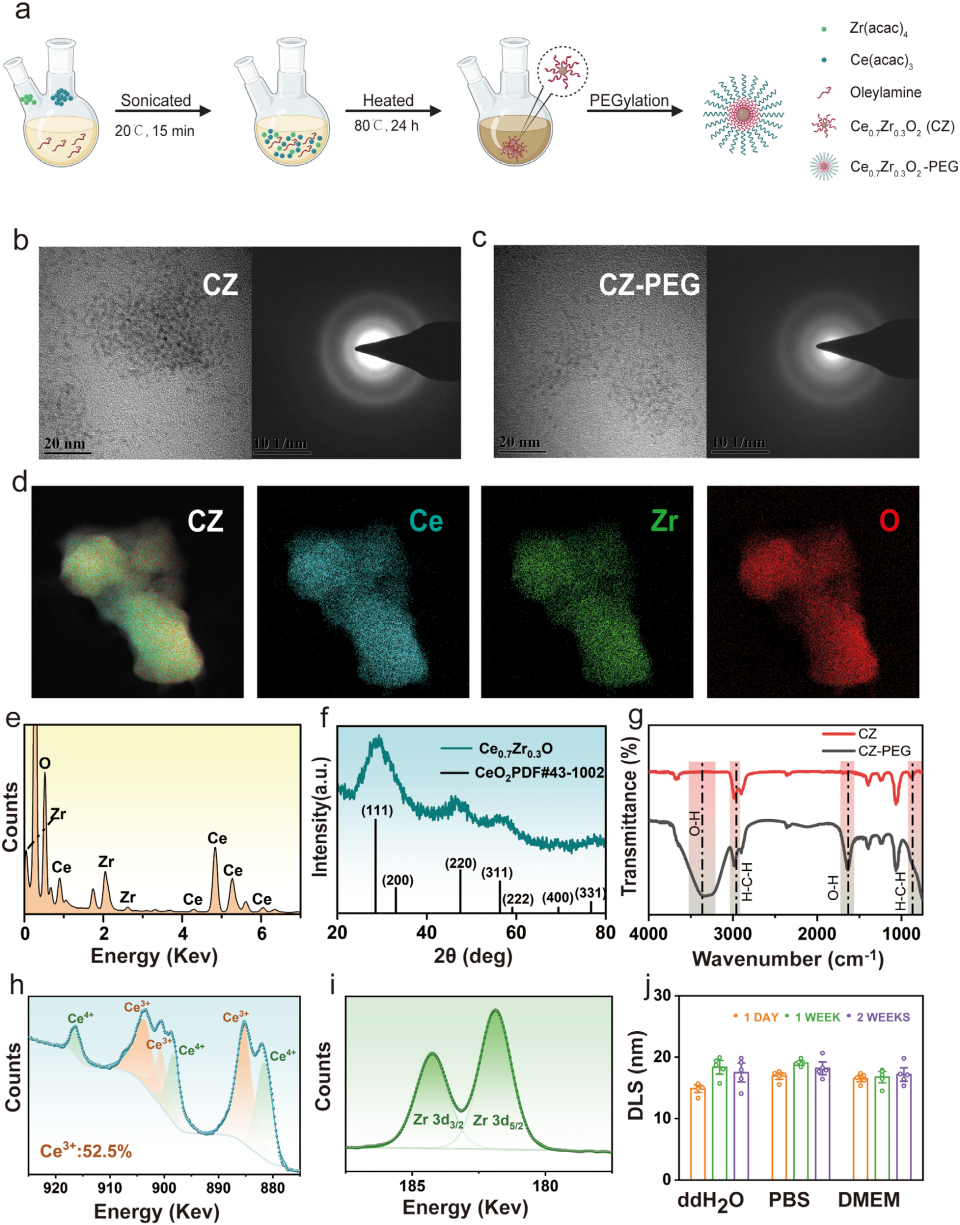
Synthesis and characterization of CZ Nanozymes. a) Scheme of the synthetic process of CZ Nanozymes. b,c) HRTEM images and SEAD of CZ Nanozymes and CZ‐PEG. Scale bar: 20 nm. d,e) STEM–EDS elemental mapping images of CZ Nanozymes. f) XRD patterns of CZ Nanozymes. The black bars represent the reference peaks of ceria. g) FTIR spectrum of CZ Nanozymes. h) XPS spectrum of the Ce in CZ Nanozymes. i) XPS spectrum of the Zr in CZ Nanozymes. j) Stability of CZ Nanozymes DLS in different physiological solutions over time (n = 5/group).

Ce_0.7_Zr_0.3_O_2_ nanozymes (CZs) were synthesized via a non‐hydrolytic sol‐gel reaction method and subsequently encapsulated with phospholipid‐PEG to enhance aqueous dispersibility and biological compatibility (**Figure** **1**a). High‐resolution transmission electron microscopy (HRTEM) revealed uniform, spherical nanoparticles with diameters of 2 nm before and after PEGylation, with preserved crystalline structure (Figure 1b,c). Energy dispersive X‐ray spectroscopy (EDS) analysis confirmed the expected Ce:Zr ratio of 67:33, and elemental mapping indicated homogeneous atomic distribution (Figure 1d,e).

Further structural validation was provided by X‐ray diffraction (XRD) showing characteristic fluorite diffraction peaks, and fourier transform infrared (FTIR), which confirmed PEG modification through O‐H and C‐H vibrational modes (Figure 1f,g). X‐ray photoelectron spectroscopy (XPS) analysis demonstrated an increased Ce^3+^ content (52.5%) upon Zr incorporation, supporting enhanced redox capacity (Figure 1h,i). Stability assessments using dynamic light scattering (DLS) and zeta potential measurements in double distilled water (ddH_2_O), phosphate buffered saline (PBS), and Dulbecco's Modified Eagle Medium (DMEM) over 14 days showed negligible variation in particle size or surface charge, indicating good colloidal stability in biological media (Figure 1j; Figure S1, Supporting Information), and similar stability was observed in plasma after 24 h (Figure S3, Supporting Information).

### 2.2 ROS‐Scavenging Activity and Enzyme‐Mimicking Activities of CZs

**Figure 2:**
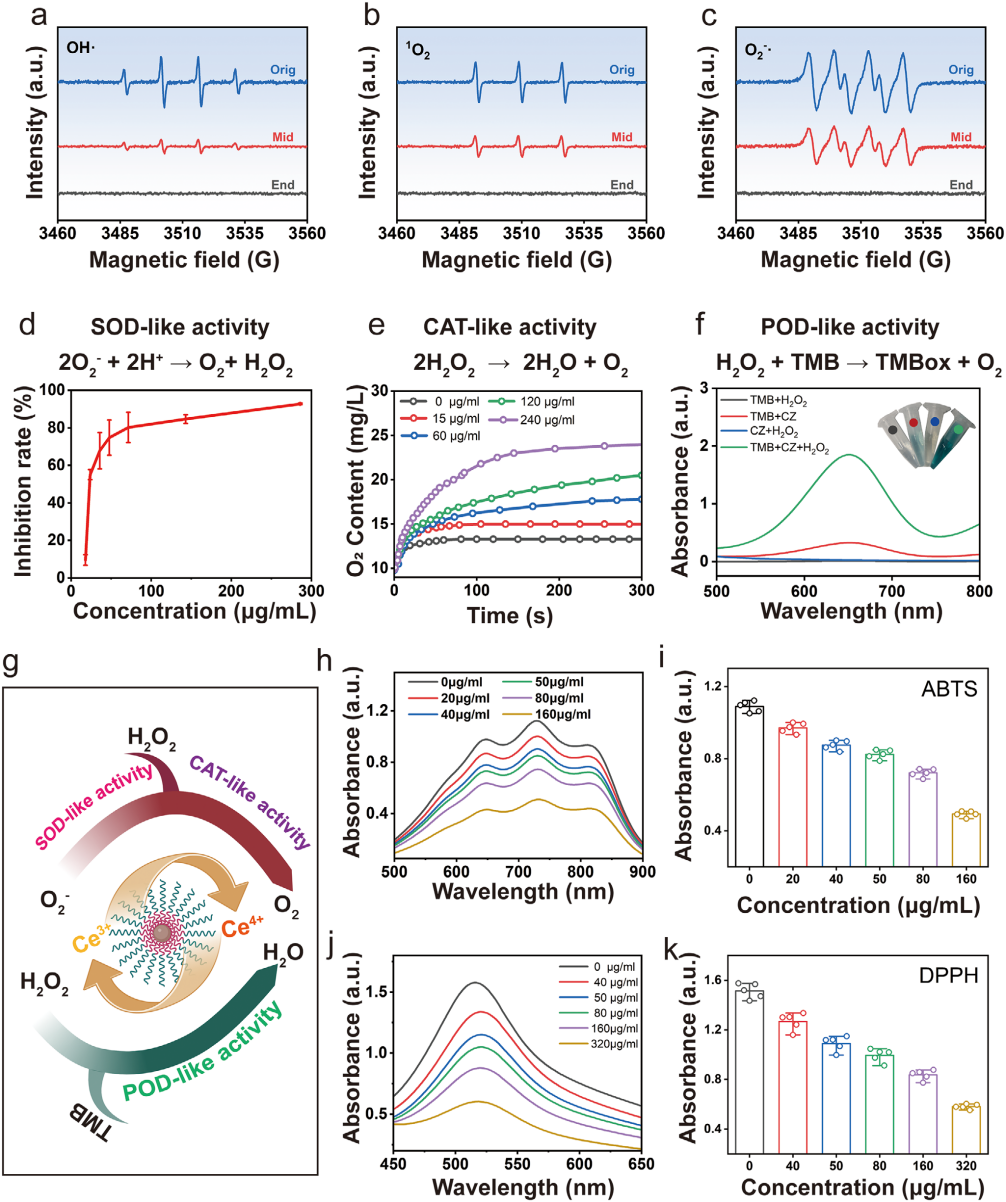
The ROS‐scavenging activity and enzyme‐mimicking activities of CZ‐PEG. ESR results of CZ‐PEG for OH· a), ^1^O_2,_ b) and O_2_^.‐^ c) elimination. d) SOD‐like activity of CZ‐PEG. e) CAT‐like activity of CZ‐PEG. f) POD‐like activity of CZ‐PEG. g) Schematic illustration of enzyme‐mimicking activities of CZ‐PEG. The scavenging activity of CZ‐PEG toward two typical free radicals was monitored via UV−vis spectroscopy and quantitatively analyzed: h,i) ABTS (n = 4/group), and j,k) DPPH (n = 4/group).

Considering the valence changes of Ce ions in CZ nanozymes and the resulting generation of oxygen vacancies, ROS scavenging is the critical component in addressing neuronal oxidative stress following TBI. The ROS‐scavenging activity of CZ nanozymes was evaluated by quantitatively assessing their effectiveness in clearing major ROS species, such as superoxide anion radical (O_2_^−^), hydroxyl radical (·OH), and singlet oxygen radical (^1^O_2_), through a standard electron spin resonance (ESR) assay. **Figure** **2**a–c demonstrates the remarkable efficacy of CZ nanozymes in rapidly eliminating these representative ROS radicals within a reaction time of only 10 min, underscoring their ability to efficiently decompose these species into non‐toxic by‐products.

Furthermore, we assessed the diverse enzymatic properties exhibited by CZ nanozymes by employing commonly used substrates for natural enzymes under physiological conditions. Superoxide dismutase (SOD), a key cellular antioxidant enzyme that neutralizes ROS, catalyzes the dismutation of O_2_^−^ into O_2_ and H_2_O_2_. As such, SOD mimetics hold promise as therapeutic agents for mitigating oxidative stress following TBI. Initially, we explored the SOD‐like activity of CZ nanozymes. The generation of O_2_^−^ in situ can be achieved through the reaction between xanthine (Xan) and xanthine oxidase (XOD). To assess the capability of CZ nanozymes in scavenging O_2_^−^, we employed 2‐(4‐iodophenyl)‐3(4‐nitrophenyl)‐5‐(2, 4‐disulfophenyl)‐2H‐tetrazolium (WST‐1). This compound, when combined with O_2_^−^, generates a colorimetric response that is measured at 450 nm. Importantly, there was a noticeable decrease in the intensity of the colorimetric signal with increasing concentrations of CZ (Figure 2d), and a distinct plateau phase was observed, indicating the efficient SOD‐like activity demonstrated by CZ.

In the subsequent investigation, we substantiated the efficacious catalase (CAT)‐like activity of CZ nanozymes. These nanozymes effectively emulate the function of CAT by catalyzing the breakdown of two H_2_O_2_ molecules into O_2_ and H_2_O. This enzymatic action serves to mitigate the accumulation of H_2_O_2,_ thereby shielding organisms from oxidative damage induced by peroxides. The CAT‐like activity of the CZ nanozymes was further confirmed by detecting the generation of O_2_ resulting from the decomposition of H_2_O_2_, facilitated by the employment of a dissolved oxygen meter. Moreover, the CAT‐like activity of CZ was corroborated by observing the release of O_2_ bubbles in solution and noting an increase in O_2_ production that correlated with escalating concentrations of CZ (Figure 2e). These empirical observations furnish conclusive evidence that CZ serve as potent analogues of CAT in the elimination of H_2_O_2_.

Peroxidase (POD) are enzymes that catalyze the decomposition of H_2_O_2_ into H_2_O and O_2,_ and serves as a key component of the endogenous antioxidant defense mechanisms in biological systems. To evaluate the POD‐like activity, the chromogenic substrate 3, 3′, 5, 5′‐tetramethylbenzidine (TMB) was selected and employed in a standardized procedure. In the presence of TMB, CZ demonstrated their catalytic prowess by facilitating the conversion of H_2_O_2_ to H_2_O, showcasing their POD‐like activity. Simultaneously, the initially colorless TMB underwent a chemical transformation into the oxidized form, known as blue oxidized TMB (TMBox), which exhibited maximum characteristic absorbance at 652 nm (Figure 2f). Conversely, control experiments lacking CZ or H_2_O_2_ did not exhibit any significant alteration in color (Figure 2f, inset). This compelling evidence substantiates the fact that CZ possesses analogous activity to natural POD enzymes. Based on these reliable experimental results, leading to the conclusion that the CZ has multi‐species bioenzymatic activities (Figure 2g).

The free radical scavenging capacity of CZ was subsequently evaluated through the implementation of multiple standard assays. To assess the free radical scavenging capacity, well‐established antioxidant activity tests such as the 2,2‐diphenyl‐1‐pyridine hydrazine radical (DPPH·) assay (Figure 2h,i) and the 2,2′‐azino‐bis(3‐ethylbenzothiazoline‐6‐sulphonate) radical ion (ABTS·+) assay (Figure 2j,k) were initially employed. Notably, CZ displayed a remarkable proficiency in scavenging radicals in both the DPPH· and ABTS·+ assays. Furthermore, the scavenging efficacy significantly increased with escalating CZ concentrations, suggesting a distinct concentration‐dependent efficacy of CZ.

### 2.3 Synergistic Effects of CZs and Nimodipine in Treating Neuronal Cell Injury

**Figure 3:**
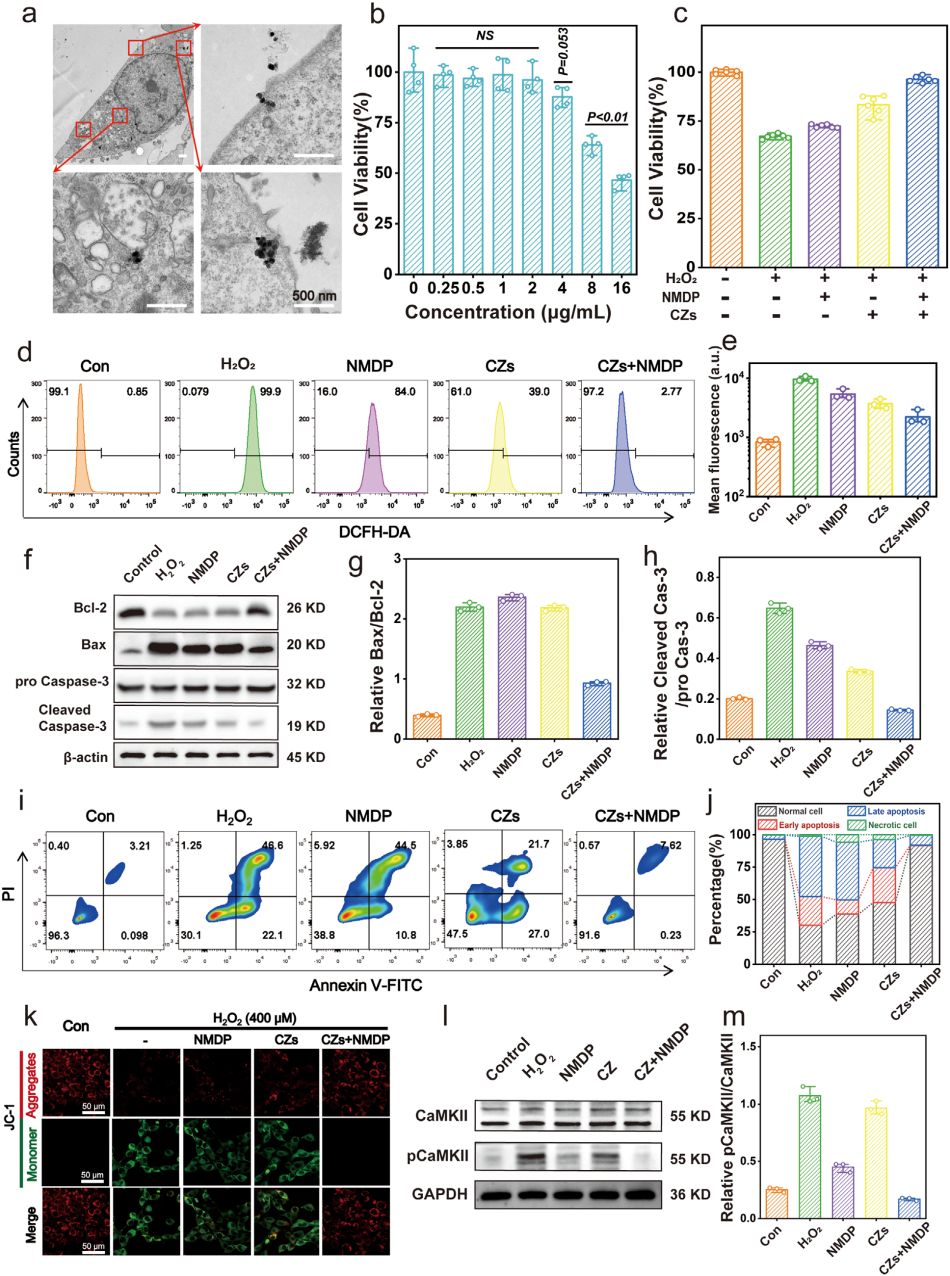
Synergistic effects of CZs and nimodipine in Treating Neuronal Cell Injury. a) Bio‐TEM images of HT22 cells after being incubated with CZs. Scale bar: 500 nm. b) Cytotoxicity of CZs toward HT22 cells (n = 4/group). c) Relative cell viabilities of HT22 cells after various treatments (n = 6/group). d,e) Flow cytometric analysis on HT22 cells stained with DCFH‐DA and the corresponding semiquantitative analysis (n = 3/group). f) Western blot analysis of Bcl‐2, Bax, pro Caspase‐3 and Cleaved Caspase‐3 in HT22 cells after varied treatments. g,h) The corresponding quantitative analysis of Bax/Bcl‐2 and Cleaved Caspase‐3/ pro Caspase‐3 protein expressions based on western blotting results (n = 3/group). i,j) Flow cytometric analysis on HT22 cells co‐stained with Annexin V‐FITC and PI and the corresponding semiquantitative analysis. k) JC‐1 staining of HT22 cells after different treatments. Scale bar: 50 µm. l,m) Western blot analysis and quantification of pCaMKII/CaMKII in HT22 cells (n = 3/group).

It is important to substantiate the efficacy of CZs in counteracting oxidative stress when combined with nimodipine at the cellular level, considering their multi‐enzyme nature and proficient free radical scavenging ability exhibited at the solution level. The investigation into the cellular uptake behavior of CZ involved a meticulous analysis utilizing biological transmission electron microscopy (TEM) images and fluorescence co‐localization techniques. Remarkably, upon 6 h of co‐incubation, discernible CZs were observed within HT22 hippocampal neuronal cells, as depicted in **Figure** **3**a. Furthermore, the progressive internalization of the nanozymes was corroborated by the augmented red fluorescence intensity observed in rhodamine B‐labeled CZs (Figure S4, Supporting Information). Subsequent to our initial investigations, we comprehensively assessed the cytotoxicity exhibited by the nanozymes using both the CCK‐8 assay and cytofluorescence analysis (Figure 3b; Figure S5, Supporting Information). Our findings revealed that a noteworthy reduction in cellular viability was observed at CZ concentrations surpassing 2 µg ml^−1^. Consequently, we adopted the concentration of 2 µg ml^−1^ as the subsequent therapeutic concentration for our in vitro experiments.

Furthermore, we sought to evaluate the in vitro efficacy of the nanozymes under diverse conditions, encompassing their application in isolation as well as in combination with nimodipine, within an oxidative stress model induced by hydrogen peroxide in HT22 cells. Remarkably, the group receiving the combined treatment exhibited the highest level of cell recovery, with cell viability increasing from 67.11% to 96.28%. (Figure 3c; Figure S6, Supporting Information). We further used flow cytometry and confocal microscopy to evaluate the scavenging of ROS caused by cellular oxidative stress. The treatment effectively mitigated oxidative stress induced by hydrogen peroxide, reducing high fluorescence intensity in 97.2% of cells and decreasing the mean fluorescence intensity (MFI) from 9625 to 2220 arbitrary units (a.u.). (Figure 3d,e; Figure S7, Supporting Information). The results in vitro were further assessed qualitatively by calcein acetoxymethyl (calcein AM)/propidium iodide (PI) assay, with green/red fluorescence observed by fluorescence microscopy (Figure S8, Supporting Information). The fluorescence results highlight the significant outcomes of the combination.

Considering the favorable antioxidative stress effect, we proceeded to substantiate its potential anti‐apoptotic capability through western blot experiments (Figure 3f). The results revealed that the synergy of CZs with nimodipine significantly reduced the expression of pro‐apoptotic proteins, as evidenced by a decrease in the Bax/Bcl‐2 ratio from 2.2 to 0.9, and efficiently inhibited caspase‐3 activation, with the cleaved caspase‐3/total caspase‐3 ratio dropping from 0.6 to 0.1 (Figure 3g,h). Following successful modeling, HT22 cells were subjected to a conventional flow apoptosis assay after staining with membrane‐bound proteins V‐FITC and PI. Figure 3i,j clearly demonstrates that the combination reduced the percentage of apoptotic cells from 68.7 to 7.85%. Notably, the administration of CZs in conjunction with nimodipine resulted in a significant reduction in both early and late‐stage apoptosis, highlighting the pronounced efficacy of this therapeutic combination.

In order to investigate the association between neuronal apoptosis and mitochondrial dysfunction, we conducted an examination of changes in mitochondrial membrane potential across different experimental groups utilizing the JC‐1 probe. The presence of JC‐1 in the form of aggregates, represented by red fluorescence, and monomers, represented by green fluorescence, indicates the polarization or depolarization state of mitochondria, respectively, corresponding to intact and disrupted mitochondrial membranes. Notably, the combined administration of CZs and nimodipine exhibited significant mitigation of mitochondrial membrane disruption, as evidenced by the observed shift from green to red fluorescence (Figure 3k).

**Figure 4:**
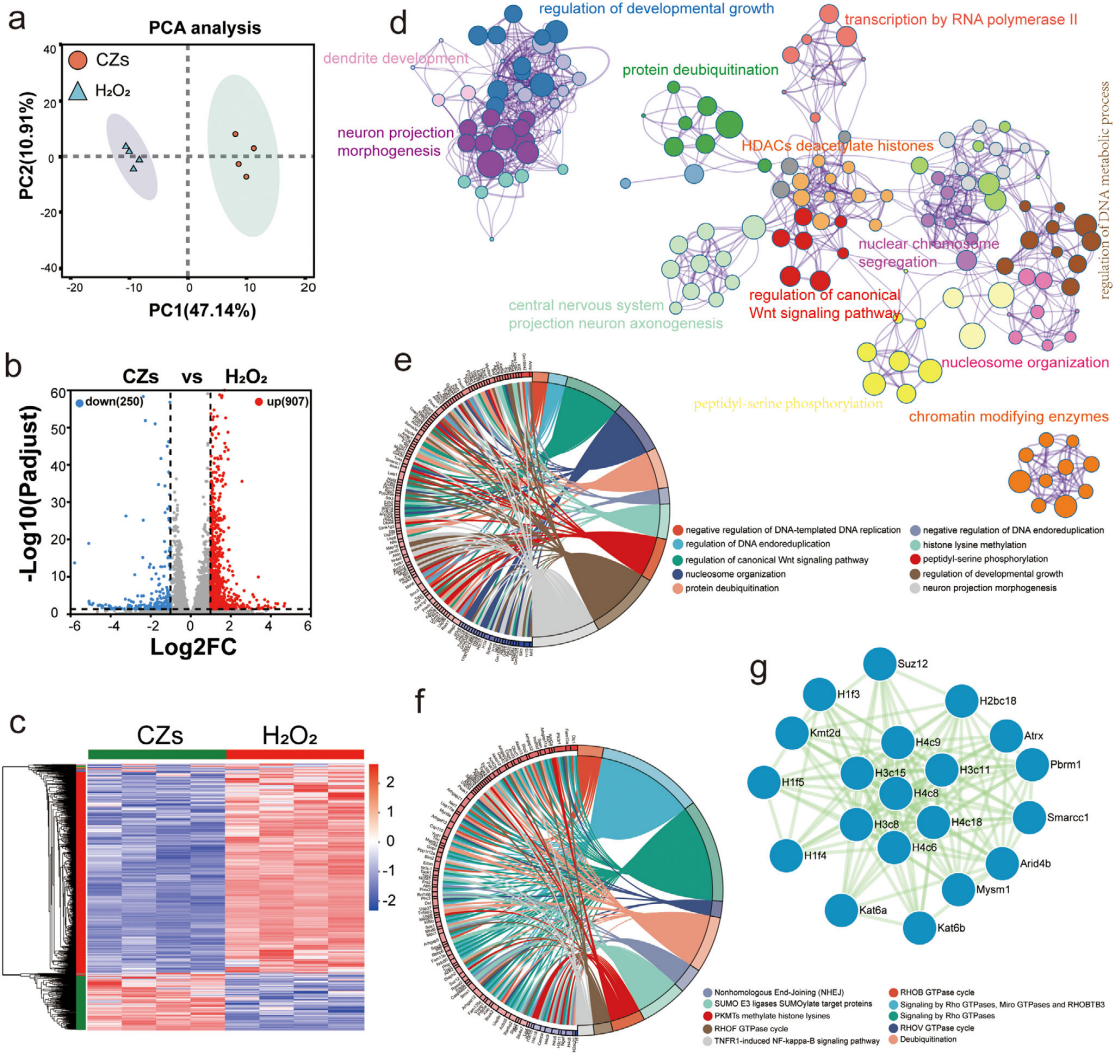
RNA sequencing of HT22 cells treated with CZs. a) PCA analysis was conducted to demonstrate the clustering of samples based on gene expression profiles. Each data point represents the PCA result for each sample. b)Heatmap of significant genes involved after CZ treatment, with fold change ≥ 2 and P < 0.05. c) Volcano plot displaying the upregulated and downregulated genes identified by CZs treatment. d) Pathway and process enrichment analysis. e) GO clustering chord diagram analyzes gene ontology and gene relationships. f) Reactome clustering chord diagram identifies key pathways and gene interactions. g) Protein‐protein interaction network of functional genes in the intersection.

Furthermore, as calcium overload represents a hallmark pathological alteration associated with HT22 neuronal cell apoptosis, we employed confocal microscopy and the fluo‐4 AM fluorescent probe to assess the efficacy of the combination treatment in regulating calcium homeostasis (Figure S9, Supporting Information). Intriguingly, through Western blot experiments, we observed that the combined application of CZs and nimodipine effectively inhibited the phosphorylation of CaMKII (Figure 3l), and the phosphorylation level of CaMKII was restored to a considerably higher level than in the control group (**Figure** **4**m). This finding suggests that the innovative drug combination holds promise for enhancing the functional recovery of neuronal cells following injury, thus laying the groundwork for future in vivo investigations.

Although CZs do not directly target calcium channels, they appear to indirectly modulate calcium signaling pathways by alleviating oxidative stress key upstream driver of calcium dysregulation in neurons. Excessive ROS production has been shown to impair mitochondrial function and compromise calcium buffering capacity, ultimately leading to CaMKII hyperactivation and excitotoxicity. By scavenging ROS and stabilizing mitochondrial membrane potential, CZs may help preserve intracellular calcium homeostasis. This hypothesis is supported by our observation of reduced CaMKII phosphorylation and decreased neuronal apoptosis in the combination therapy group. These findings suggest a mechanistic link between CZs‐mediated oxidative stress attenuation and the indirect suppression of calcium overload‐associated signaling cascades in TBI pathology.

### 2.4 Potential Mechanism of CZs for the Mitigation of Oxidative Stress in Neuronal Cells

To elucidate the efficacy of CZs nanozymes and investigate the potential mechanisms underlying their neuroprotective effects, mRNA sequencing was performed to identify differences between hydrogen peroxide‐induced injury and CZs group‐treated HT22 hippocampal neuronal cells. Unsupervised principal component analysis (PCA) revealed distinct differences between the various treatment groups (Figure 4a). Cluster analysis clearly separated differentially regulated genes between the control and CZs‐treated groups. A total of 1157 differentially expressed genes were identified between the CZs‐treated and H_2_O_2_‐impaired groups, with 250 up‐regulated and 907 down‐regulated genes (Figure 4b,c).

Initially, we conducted a comprehensive enrichment analysis of the genes involved in specific pathways and processes (Figure 4d). The outcomes of this analysis predominantly highlighted significant enrichment in pathways associated with the regulation of developmental growth, biological processes related to protein deubiquitination, the morphogenesis of neuron projections, and the axonogenesis of central nervous system projection neurons. Gene Ontology (GO) analysis highlighted their involvement in processes such as the negative regulation of DNA‐templated DNA replication, regulation of intra‐DNA replication, developmental growth regulation, and neuronal synaptic morphology (Figure S11a, Supporting Information; Figure 4e). Reactome pathway enrichment analyses (Figure S11b, Supporting Information; Figure 4f) revealed that pathways such as the RHOB GTPase cycle, signaling by Rho GTPases, Miro GTPases, and RHOBTB3, as well as the RHOV GTPase cycle, are closely linked to the therapeutic mechanisms of CZs nanomedicines. Rho GTPases not only play a pivotal role in cytoskeletal reorganization but also regulate various signaling pathways associated with oxidative stress. For example, Rho GTPases modulate cellular stress responses by activating NADPH oxidase, which produces ROS.^[^ ^35^ ^]^ Additionally, Miro GTPase and RHOBTB3 are deeply involved in mitochondrial function,^[^ ^36^ ^]^ a central hub in both the production and consequences of oxidative stress.

To further elucidate the underlying mechanisms, we conducted Gene Set Enrichment Analysis (GSEA) on the entire gene set (Figures S12 and S13a,b,d, Supporting Information). The results revealed significant enrichment in pathways related to nuclear protein‐containing complexes, chromosome‐associated proteins, and protein‐DNA complexes (Figure 4g). Given the critical role of these DNA‐related processes in cellular function, we propose that the protective effects of CZs may be closely linked to the inhibition of neuronal apoptosis. Additionally, the enrichment observed in the catalytic complex pathway (Figure S13c, Supporting Information) suggests that nanozymes may be actively involved in enzymatic reactions and serve as key components in cellular metabolism and signaling pathways. To verify the link between ROS and calcium signaling, we performed live‐cell calcium imaging. CZs significantly reduced H_2_O_2_^−^induced calcium overload, lowering Fluo‐4 fluorescence from 56.32 ± 18.03 to 14.92 ± 3.79 a.u. (Figure S9, Supporting Information). Conversely, calcium influx induced by ionomycin elevated ROS levels, which were also suppressed by CZs (Figure S10, Supporting Information). These results underscore the critical role of CZs in protecting neuronal cells from oxidative stress‐induced damage.

### 2.5 Synergistic Effects of CZs and Nimodipine in the Treatment of Mice After TBI

**Figure 5:**
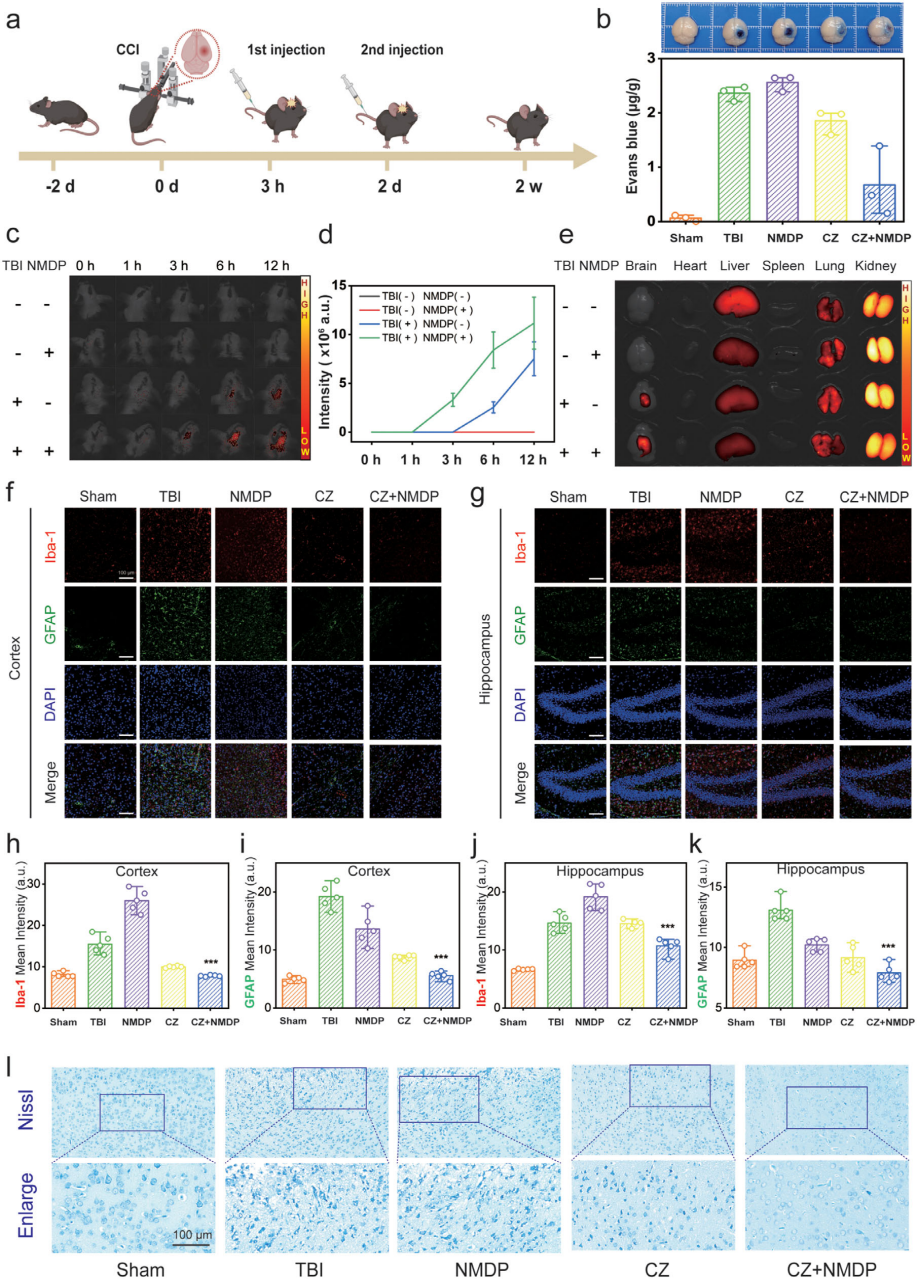
Synergistic effects of CZs and nimodipine in the treatment of mice after TBI. a) Schematic diagram of the process in vivo. b) Representative images and quantitative analysis of Evans blue leakage (n = 3/group). c,d) Fluorescent images after various groups in intravenously injected TBI mice, and quantification of DiR intensity in the brain 12 h (n = 3/group). e) Fluorescent images of tissue distribution in intravenously injected TBI mice. In the cortex f,h,i) and hippocampus g,j,k), astrocytes were labeled with GFAP (green), microglia with Iba‐1 (red), and nuclei with DAPI (blue). i) Representative pictures showing the results of Nissl staining after various treatments. Scale bar: 100 µm. The levels of significance were set at *p* < 0.05 (^*^), *p* < 0.01 (^**^), and *p* < 0.001 (^***^). n.s. = non‐significant.

To further investigate the cerebroprotective effects of CZs and nimodipine in an in vivo setting, the well‐established controlled cortical impact (CCI) model was utilized to simulate TBI, and experimental procedures were conducted accordingly (**Figure** **5**a). The aforementioned results have demonstrated that TBI induces dysfunction and disruption of the BBB. ROS have been implicated in exacerbating BBB injury. Through previous investigations, we have confirmed the ability of CZs to scavenge ROS. To explore the combined effects of these drugs on the integrity of the BBB, we employed the Evans blue extravasation assay to assess BBB permeability following TBI. To evaluate the therapeutic efficacy of our approach post‐TBI induction, we initiated a treatment regimen immediately and evaluated outcomes after 24 h. As shown in Figure 5b, the combination treatment resulted in the lowest BBB permeability at 0.67 µg g^−1^, significantly outperforming monotherapy with CZs (1.85 µg g^−1^), nimodipine (2.56 µg g^−1^), and the TBI control group (2.36 µg g^−1^). These findings highlight the efficacy of the combination therapy in maintaining BBB integrity. Concurrently, brain water content was measured to evaluate cerebral edema following TBI. Figure S15, Supporting Information (Supporting Information) shows that combined therapy reduced cerebral edema to 73.20% from 79.58%, while nimodipine alone had no significant effect, with edema at 79.88%.

These observations imply that nimodipine may facilitate the delivery of CZs to the brain. To validate this hypothesis, we conducted an in vivo fluorescence imaging study (Figure 5c,d). The study revealed that the combined group exhibited an earlier accumulation of nimodipine in the brain within the first hour. In contrast, the group treated with nanozymes alone showed significant accumulation only after 3 h. Moreover, the fluorescence intensity of the combined group was consistently superior to that of the group treated with nanomaterials alone throughout the 12‐h experimental duration. These results suggest that nimodipine enhances the intracerebral accumulation of nanozymes. However, the mechanism remains to be fully elucidated. Additionally, renal metabolism was identified as the primary route for nanozyme clearance (Figure 5e). We selected a low dose of nimodipine (1 mg kg^−1^) based on previous reports^[^ ^37^ ^]^ indicating its transient and dose‐dependent effect on BBB permeability. Consistently, our findings suggest that this dosing strategy facilitates passive accumulation of co‐administered agents in injured brain regions without inducing prolonged barrier disruption or systemic side effects.

Experimental and clinical findings suggest that cognitive deficits induced by TBI are associated with alterations in the traumatized cortex and hippocampus. Therefore, to evaluate the therapeutic effectiveness of the combined administration of CZs and nimodipine following TBI, we performed a neuroinflammation evaluation through immunostaining for ionized calcium‐binding protein 1 (Iba‐1), a marker linked to activated microglia. Additionally, the expression of glial fibrillary acidic protein (GFAP), a prototypical indicator of reactive astrocytes, was notably elevated following TBI. Notably, the levels of Iba‐1 and GFAP expression were significantly heightened in the untreated TBI and nimodipine‐treated groups in comparison to the sham‐operated group, suggesting that nimodipine alone did not mitigate neuroinflammation. In contrast, the co‐treatment with CZs and nimodipine led to a significant downregulation of Iba‐1 and GFAP expression, suggesting an augmented therapeutic effect of nimodipine when paired with nanomaterials (Figure 5f,h,i). The cortex adjacent to the TBI site in the co‐treated group exhibited the lowest levels of Iba‐1 and GFAP, indicating a synergistic anti‐inflammatory response. Specifically, the combined therapy resulted in a reduction of mean fluorescence intensity (MFI) for Iba‐1 from 15.45 to 7.81 a.u. and for GFAP from 19.22 to 5.57 a.u. Moreover, the co‐treatment demonstrated consistent anti‐inflammatory effects in the hippocampus (Figure 5g,j,k), with MFI reductions for Iba‐1 from 14.63 to 10.70 a.u. and for GFAP from 13.10 to 7.89 a.u. These data suggest that the combination of CZs and nimodipine exerted the most potent inhibitory effect on neuroinflammation among all treatment modalities.

To evaluate the functional status of neurons following TBI, we employed Nissl staining to visualize neuronal Nissl bodies in the cortical injury area (Figure 5i). In the sham group, neurons exhibited intact morphology with distinct, dark blue Nissl bodies clearly visible in the cytoplasm. Conversely, in the post‐traumatic cortical injury area, neurons displayed crumpling and deformation, and selective staining of damaged neurons was compromised due to cell membrane rupture. Treatment with CZs alone partially restored neurons within the cortical injury zone. Notably, the combined treatment group demonstrated a more pronounced neuroprotective effect, evidenced by more intact neuronal myelin sheaths in the corpus callosum, as revealed by Luxol Fast Blue (LFB) staining (Figure S16, Supporting Information). These results indicate that the combination of CZs and nimodipine effectively mitigates neuronal damage in the post‐traumatic cortical injury area. Furthermore, assessment using Fluoro‐Jade B (FJB) fluorescence staining revealed a significant presence of degenerated neurons (indicated by green fluorescence) in the TBI group. Remarkably, the combination treatment group demonstrated a substantial alleviation of neuronal degeneration, nearly reaching levels comparable to those observed in the sham‐operated group (Figure S17, Supporting Information). Collectively, these findings indicate that CZs combined with nimodipine also have a satisfactory anti‐degenerative effect on neurons in the TBI mouse model, which is consistent with the results of the HT22 cell experiments.

### 2.6 Synergistic Effects of CZs and Nimodipine in Improving Long‐Term Neurologic Function After TBI in Mice

**Figure 6:**
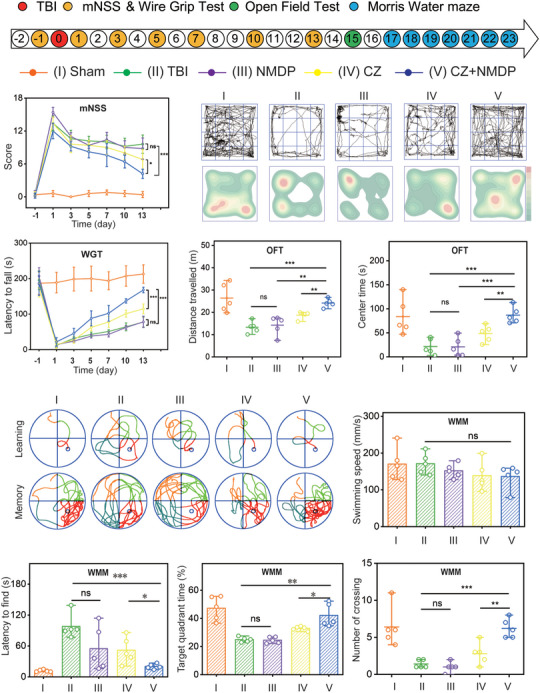
Synergistic effects of CZs and nimodipine in improving long‐term neurologic function after TBI in mice. a) Time course diagram and experimental design of mouse experiments. b) The mNSS scores were calculated after various treatments (mean±SD). c) Images of open field test trajectories and heat maps for assessing locomotor behavior. d) Motor function was evaluated by the hanging wire grip test (mean±SD). e,f) Total distance and center time for each group in the open field test. g,h) Representative images of the swimming trajectories during the learning phase and memory phase. The latency, i) target quadrant time, j) and crossing number, k) of the Morris water maze. l) Swimming speed of mice in each group (n = 5/group). Statistical significance was analyzed using one‐way analysis of variance (ANOVA). The levels of significance were set at *p* < 0.05 (^*^), *p* < 0.01 (^**^), and *p* < 0.001 (^***^). n.s. = non‐significant.

Lastly, a comprehensive series of behavioral experiments was meticulously conducted to elucidate the impact of the combined treatment regimen comprising CZs and nimodipine on long‐term neurological function post‐TBI. An elaborate experimental design was formulated (**Figure** **6**a), and meticulous data recording was undertaken to ensure robust presentation of the outcomes. Subsequent to the in vivo experimental grouping, we proceeded to conduct behavioral experiments evaluating the modified neurological severity scores (mNSS) as a measure of neurological function. In context, while the co‐treatment group did not achieve the baseline levels of the sham‐operated cohort, it nonetheless exhibited a pronounced therapeutic benefit compared to other control groups. As illustrated in Figure 6b, this synergistic treatment resulted in a substantial decrease from 12.6 to 4.2, which was significantly more favorable than the reduction observed in the TBI group, which improved from 13.4 to 9.6.

To assess the impact of the combination treatment on motor function, we employed a wire grip test (WGT) from day 1 before TBI until day 13 post‐TBI. Our findings revealed that the scores exhibited a gradual increase for all groups, except the Sham group, as the number of days following TBI increased. This observation suggests a progressive recovery of motor dysfunction in the mice over time (Figure 6d). Notably, the co‐treatment group scored significantly higher after TBI compared to the group treated with combination therapy, reaching a grasped time of 168.0±7.8 s, significantly due to 77.8±13.9 s in the TBI group, which implies that the combination therapy exerted the strongest influence in promoting motor function recovery.

Subsequently, on the 15th day post‐TBI, we conducted the open field test (OFT) to evaluate the voluntary locomotor and exploratory capacities of the mice. As depicted in Figure 6c, the first row illustrates the representative trajectory graphs of mice belonging to distinct subgroups, while the second row exhibits corresponding heat maps reflecting the distances covered. The findings showed that the co‐treatment group not only demonstrated substantial recovery, approaching a behavioral distance similar to that of the sham‐operated group, but also showed a higher proportion of centripetal time of 86.9±21.5 s (Figure 6e,f), especially compared to the TBI group of 17.2±16.5 s. This suggests enhanced exploratory ability within this group of mice, thus underscoring the efficacy of co‐treatment treatment in alleviating TBI‐induced motor function impairment and exploratory deficits.

Subsequently, we employed the Morris water maze (MWM) test to evaluate the mice's spatial learning and memory capabilities. Following TBI, a noticeable decline in learning performance was observed, as evidenced by significantly prolonged time to reach the plateau compared to the sham‐operated group (Figure 6g). Remarkably, treatment with CZs alone resulted in enhanced learning ability relative to the TBI group. However, in the co‐treatment group, learning ability was preserved to a greater extent, while the escape latency was further reduced, from 97.9 ± 20.2 to 23.8 ± 5.0 s in the TBI group (Figure 6i).

To assess spatial memory post‐TBI, the hidden platform was removed on the 23^rd^ day, allowing for an examination of the mice's performance (Figure 6h). Notably, the co‐treatment administration markedly enhanced spatial memory capabilities, as demonstrated by an extension in the retention time within the target quadrant, increasing from 25.0% to 42.2% (Figure 6j), alongside a significant increase in both platform searches and crossings (Figure 6k). It is worth mentioning that the swimming speed of mice among all groups during the Morris water maze test exhibited comparability (Figure 6l). These results suggest a potential relationship between the observed outcomes in the Morris water maze test and the drug combination's ability to mitigate TBI‐induced hippocampal damage. Collectively, these extensive in vivo long‐term neurobehavioral assessments demonstrate that the combination treatment of CZs and nimodipine significantly ameliorates neurological dysfunction induced by TBI.

To further assess the translational relevance of the proposed therapy, we compared the combination of CZs and nimodipine with edaravone, a clinically approved neuroprotective agent widely used in the treatment of acute ischemic and TBI. As shown in Figure S18 (Supporting Information), the co‐treatment group exhibited fewer FJB‐positive degenerating neurons and smaller edema volumes on T2‐weighted MRI compared to the edaravone group, indicating efficacy in mitigating neuronal degeneration and brain swelling. To complement behavioral assessments and provide objective imaging‐based evidence of neuroprotection, we performed T2‐weighted MRI at 28 days post‐injury. As shown in Figure S19 (Supporting Information), mice receiving the co‐treatment group exhibited smaller damaged areas on T2‐weighted images compared to other groups. To further validate the long‐term therapeutic efficacy, we incorporated Morris water maze testing (Figure S20, Supporting Information), which revealed significant cognitive improvements in the combination group.

### 2.7 Toxicity Evaluation

Finally, the biosafety of CZs was rigorously assessed. For short‐term hemocompatibility, we conducted in vitro hemolysis experiments using various concentrations of nanozymes. Based on in vitro cytoprotection and in vivo dose‐escalation safety data, a low‐dose regimen of 2 µg mL^−1^ (in vitro) and 2 mg kg^−1^ (in vivo, i.v.) was selected, which demonstrated significant neuroprotective effects without observable toxicity (Figure S14, Supporting Information). The results indicated that no significant hemolysis of erythrocytes occurred at the therapeutic concentration of 2 µg mL^−1^ (Figure S21, Supporting Information). To elucidate long‐term effects, we analyzed blood samples following tail vein injection. Compared to the TBI group, the liver function indices (AST, ALT, ALP) and kidney function indices (CREA, BUN, UA) in the CZs and co‐treatment groups remained within normal ranges. This suggests that there were no adverse effects on liver and kidney functions, even under conditions of TBI (Figure S22, Supporting Information). Furthermore, histopathological examination of vital organs from experimental mice, using H&E staining, revealed no significant tissue damage or morphological changes, indicating minimal biotoxicity of the nanomaterials (Figure S23, Supporting Information). To evaluate potential chronic toxicity, we extended the histopathological assessment of major organs to 42 days post‐treatment. As shown in Figure S24 (Supporting Information), H&E‐stained sections of the heart, liver, spleen, lung, kidney, and brain revealed no detectable signs of inflammation, fibrosis, necrosis, or other pathological alterations across all treatment groups. In summary, these comprehensive findings provide compelling evidence that CZs exhibit minimal systemic toxicity, thereby supporting their potential for clinical application. To verify the reproducibility of CZs synthesis, catalytic activity and ABTS scavenging efficiency were evaluated across three independent batches (Figure S2, Supporting Information), demonstrating good batch‐to‐batch consistency.

## 3 Conclusion

In summary, we designed and synthesized ultrasmall catalytic Ce_0.7_Zr_0.3_O_2_ nanozymes (CZs) with the aim of exploring their neuroprotective effects when used in conjunction with nimodipine. This study demonstrates the synergistic therapeutic potential of CZs and nimodipine in TBI, with improved efficacy compared to nimodipine monotherapy. CZs exhibit exceptional ROS scavenging ability at minimal concentrations, while nimodipine enhances brain accumulation. Their combined action effectively reduces oxidative stress, preserves mitochondrial function, and inhibits neuronal apoptosis. This strategy not only improves therapeutic outcomes but also minimizes neurotoxicity. By integrating nanomaterials with conventional pharmacotherapy, this study provides an innovative approach to TBI treatment and lays the groundwork for future translational applications.

## 4 Experimental Section

### Chemicals

Cerium (III) acetylacetonate hydrate and zirconium (IV) acetylacetonate were purchased from Sigma Aldrich. Oleylamine was obtained from Aladdin. Acetone, chloroform, and H_2_O_2_ (30%) were sourced from Shanghai University. FITC was supplied by Shanghai Runcheng Bio‐tech Co., mPEG(2000)‐PE, DSPE‐PEG (2000)‐amine, and Cy5.5 NHS ester were purchased from Rui Xi Biology. SOD assay kit, CCK‐8, DAPI, DCFH‐DA, JC‐1, and Fluo 4‐AM were obtained from Beyotime Institute of Biotechnology. Annexin V‐FITC/PI Apoptosis Detection Kits were purchased from Yeasen.

### Synthesis of Ceria‐Zirconia Nanozymes (CZ) and Phospholipid‐Polyethylene Glycol (PEG)‐Encapsulated CZ Nanozymes (CZs)

Ceria–zirconia (CZ) nanozymes were synthesized by adding a 0.5 g mixture of hydrated cerium and zirconium acetylacetonate, in the correct molar ratio, to 15 mL of oleylamine. This mixture was sonicated at 20 °C for 15 min, then heated to 80 °C at a rate of 2 °C per minute and aged at this temperature for 24 h before being cooled to room temperature. The mixture was washed several times with acetone at 5000 rpm, and the resulting CZ precipitate was dispersed in chloroform to a concentration of 10 mg mL^−1^. For the synthesis of phospholipid‐polyethylene glycol (PEG)‐encapsulated CZ nanozymes, the CZ nanomedicines in chloroform were encapsulated with phospholipid‐PEG. This involved mixing 5 mL of the chloroform CZ solution with 10 mL of chloroform mPEG (2000)‐PE. The mixture was then spin evaporated using a rotary evaporator and incubated in a vacuum oven at 70 °C for 2 h to remove the chloroform. After adding 5 mL of deionized water, the sample was filtered through a 0.4 µm filter and ultracentrifuged to remove excess mPEG (2000)‐PE. The final purified samples were dispersed in deionized water.

### Characterization of CZ and CZs

A dispersion of the CZ nanozymes was deposited onto a carbon‐coated copper grid, dried at room temperature, and then analyzed using TEM and STEM at 200 kV with a JEM‐2100f microscope (JEOL, Japan). Energy‐dispersive X‐ray spectroscopy (EDS) was conducted using a single drift detector (X‐MaxN, Oxford Instruments, UK). The hydrodynamic diameter of the samples was measured by dynamic light scattering (DLS) using a Zetasizer Nano‐ZS system (Malvern Instruments Ltd., UK). Zeta potential was determined in water using a Mastersizer 3000 laser diffraction particle size analyzer (Malvern, UK). Fourier transform infrared spectroscopy (FTIR) was performed with an FTIR‐8300 series spectrometer (Shimadzu, Japan) for spectral analysis. X‐ray photoelectron spectroscopy (XPS) was conducted using an ESCALAB 250 Xi Mg X‐ray source (Thermo Scientific, Japan). X‐ray diffraction (XRD) measurements were obtained with a D8 Advance diffractometer (Bruker, Germany).

### Antioxidant Testing via ESR

Experiments were conducted at ambient temperature using a Bruker EMX ESR spectrometer. The scavenging of ·OH, ^1^O_2,_ and O_2_^−^· by CZ was quantified using ESR spectroscopy (JEOL FA200), with DMPO serving as the spin trapping agent at a concentration of 100 mM. The radicals were identified by their characteristic electron paramagnetic resonance (EPR) signal patterns: ·OH exhibits a distinct 1:2:2:1 signal, O_2_· shows four large and two small peaks, and ^1^O_2_ displays a characteristic 1:1:1 signal.

### SOD Mimetic Activity Assay

The SOD‐like activity of CZs was evaluated using a total SOD activity assay kit based on the WST‐8 method (Beyotime, China), adhering to the manufacturer's protocol. Absorbance measurements were taken at 450 nm using a microplate reader.

### CAT Mimetic Activity Assay

The CAT‐like activity of CZs was determined by measuring the production of oxygen using a JPB‐607A portable dissolved oxygen meter (INESA Scientific Instruments, Shanghai, China). Specifically, 2 mL of CZs solutions at varying concentrations were combined with 8 mL of PBS containing H_2_O_2_, or 2 mL of CZ nanomedicine solutions were mixed with 8 mL of PBS with different concentrations of H2O2. The reaction was carried out at room temperature for 20 min, with oxygen production measured every minute.

### POD Mimic Activity Assay

The POD‐like activity of CZs was assessed using TMB as the substrate in the presence of H_2_O_2_. In a typical procedure, a solution containing TMB, H_2_O_2_, and CZs was prepared in PBS. After a specified reaction time, the absorbance was recorded using a UV–vis spectrophotometer and microplate reader, with measurements taken at 652 nm.

### ABTS and DPPH Decolorization Assays

In the ABTS assay, 7.4 mmol of ABTS was reacted with 2.6 mM potassium persulfate to generate ·ABTS+ radicals, which were stored in the dark for 24 h. The reaction with varying concentrations of CZs was monitored for 5 min, and the inhibition rate of ·ABTS+ radicals was determined by measuring the absorbance at 734 nm using UV–vis spectroscopy. In the DPPH assay, 3 mmol of DPPH was dissolved in anhydrous ethanol and reacted with different concentrations of CZs for 30 min. The inhibition rate of DPPH radicals was calculated by measuring the absorbance at 517 nm.

### Cell Culture

The HT22 (hippocampal neuronal cell line) was obtained from the Cell Bank of the Shanghai Institute of Biochemistry and Cell Biology, Chinese Academy of Sciences. The cells were maintained in high‐glucose DMEM medium, enriched with 10% fetal bovine serum and 1% penicillin/streptomycin, under a humidified environment at 37 °C with 5% CO_2_.

### Bio‐TEM Analysis

HT22 cells were plated in 6‐well plates and grown for 24 h until 70–80% confluent. They were then exposed to treatments for 24 h. The cells were collected, centrifuged, and resuspended in 2.5% glutaraldehyde fixative overnight. Following fixation in osmium tetroxide for 1 h at room temperature, cells were dehydrated and embedded in resin. Sections were imaged using TEM.

### Cell Uptake Analysis

HT22 cells were seeded in confocal glass trays at a density of 10^5^ cells per disc. The cells were then incubated with a fresh culture medium containing rhodamine B‐labeled CZ for various durations, replacing the standard cell culture medium. After incubation, the treated HT22 cells were washed twice with PBS and subsequently examined using CLSM.

### In Vitro Cytotoxicity Assay

HT22 cells were seeded in 96‐well plates and allowed to grow for 24 h. Once they reached 80% confluence, oxidative stress was induced using 400 µM H_2_O_2_. The cells were then treated with fresh media containing varying concentrations of CZs. The cytotoxicity was subsequently assessed using the CCK‐8 assay.

### In Vitro ROS Rescue Detection

HT22 cells were seeded in confocal glass trays at a density of 10^5^ cells per dish and cultured until they reached 80% confluence. An oxidative stress state was induced using H_2_O_2_. The cells were then subjected to the following treatment groups: 1. control group (no H_2_O_2_); 2. oxidative stress group (with H_2_O_2_); 3. drug‐treated group (nimodipine, NMDP); 4. nano‐enzyme group (CZs); 5. combined treatment group (NMDP + CZs); 6. nimodipine plus CZs group without H_2_O_2_. The cells were co‐incubated at 37 °C overnight, then DCFH‐DA (10 µM) was added, followed by an additional 30 min incubation, keeping away from light in the incubator. After the treatments, the cells were rinsed twice with PBS and observed using CLSM or FCM.

### Detection of Live/Dead Cells and Apoptosis Analysis

HT22 cells were seeded in confocal glass trays at a density of 10^5^ cells per dish and cultured until they reached 80% confluence. An oxidative stress state was induced using H_2_O_2_. The cells were then subjected to the following treatment groups: 1. control group (no H_2_O_2_); 2. oxidative stress group (with H_2_O_2_); 3. drug‐treated group (nimodipine, NMDP); 4. nano‐enzyme group (CZs); 5. combined treatment group (NMDP + CZs); 6. nimodipine plus CZs group without H_2_O_2_. After co‐incubation at 37 °C for 8 h, and the cells were co‐incubated with Calcein AM and PI for 30 min. After the treatments, the cells were rinsed twice with PBS and observed using CLSM or FCM.

### Detection of Mitochondrial Membrane Potential

HT22 cells were seeded in confocal glass trays at a density of 10^5^ cells per dish and cultured until they reached 80% confluence. An oxidative stress state was induced using H_2_O_2_. The cells were then subjected to the following treatment groups: 1. control group (no H_2_O_2_); 2. oxidative stress group (with H_2_O_2_); 3. drug‐treated group (nimodipine, NMDP); 4. nano‐enzyme group (CZs); 5. combined treatment group (NMDP + CZs); 6. nimodipine plus CZs group without H_2_O_2_. Then, HT22 cells were stained with JC‐1 staining using the Mitochondrial Membrane Potential Assay Kit (Beyotime‐C2006, Shanghai, China) and observed by CLSM.

### Detection of Intracellular Ca^2+^ Concentration

HT22 cells were seeded in confocal glass trays at a density of 10^5^ cells per dish and cultured until they reached 80% confluence. An oxidative stress state was induced using H_2_O_2_. The cells were then subjected to the following treatment groups: 1. control group (no H_2_O_2_); 2. oxidative stress group (with H_2_O_2_); 3. drug‐treated group (nimodipine, NMDP); 4. CZs group; 5. combined treatment group (NMDP + CZs); 6.nimodipine plus CZs group without H_2_O_2_. Then, HT22 cells were stained with Fluo‐4 AM using the Calcium Fluorescent Probe Kit (Beyotime‐S1060, Shanghai, China) and observed by CLSM.

### Western Blot Analysis

Cells from various treatment groups were rinsed with PBS and lysed to extract proteins. PVDF membranes were blocked with 5% skimmed milk for 1 h at room temperature on a decolorizing shaker. The membranes were incubated with primary antibodies against Bcl‐2 (A19693, ABclonal), Bax (A12009, ABclonal), pro caspase‐3 (ab32150, abcam), Cleaved Caspase‐3 (ab32042, abcam), CaMKII (A0198, ABclonal), and p‐CaMKII (AP1386, ABclonal), overnight at 4 °C. They were then washed three times in the decolorizing shaker. Following this, the membranes were incubated with the secondary antibody for 30 min and washed again three times. Electro chemiluminescent reagent was added and allowed to react for 1–2 min. The membranes were exposed in a dark room, and protein expression was quantified using ImageJ.

### Animals

The animal protocols were approved by the Committee on Ethics of Medicine, Naval Medical University (No. 2024SL274). Male C57BL/6 mice were acquired from Regenda Technology Co. (Shanghai, China). To minimize biological variability in treatment response, all animals used in this study were age‐matched (10 weeks), weight‐matched (22–25 g), and randomly assigned to treatment groups. All mice were housed under standardized conditions (12‐h light/dark cycle, 22± 2 °C, 50 ± 10% humidity), with ad libitum access to food and water. Healthy mice were selected for the experiments after thorough examination. The mice were divided into six groups: 1) Sham group: only the cranial foramen was opened without inducing trauma. 2) TBI group: was induced using controlled cortical impact (CCI). 3) NMDP group: nimodipine treatment only. 4) CZ group: treatment with CZs alone (2 mg kg^−1^, intravenous). 5) NMDP+CZ group: Combination treatment with nimodipine plus CZs.

### Assessment of BBB Integrity

A 2% Evans Blue solution was injected into mice via the tail vein. After allowing for circulation, the brain was perfused with saline and divided into left and right hemispheres. The injured hemisphere was weighed, cut into pieces, and incubated in dimethylformamide (1 ml/100 mg) at 60 °C for 24 h. The mixture was centrifuged at 1000 r min^−1^ for 5 min, and the absorbance was measured at 620 nm using a spectrophotometer. The Evans Blue content was then calculated using a standard curve.BBB was assessed at 24 h post‐injury, corresponding to the peak of barrier disruption in TBI models.

### Brain Water Content

Anesthetize the mice, extract their brains, and bisect them along the midline into left and right hemispheres. Weigh the injured hemisphere to get the wet weight (Ww), then dry the brain tissue in a 110 °C electric oven for 24 h until the weight stabilizes, and measure the dry weight (Wd). Calculate the brain water content using the formula: ((W_w_ – W_d_)/W_w_*100%).

### In Vivo Imaging in Mice

CZs‐Cy5.5 was administered to mice with trauma via tail vein injection. At various time points following the injection, in vivo imaging was performed to assess its accumulation in the brain. Twelve hours after the intravenous injection, the major organs of the mice, including the heart, liver, spleen, lungs, kidneys, and brain, were harvested for in vivo imaging to examine the distribution of CZs.

### TBI Model

Mice were positioned prone in a stereotaxic apparatus with their heads fixed. After disinfection, a 1 cm longitudinal incision was made along the midline to expose the skull. A 2‐mm cranial window was drilled over the right parietal bone. The dura mater was exposed, and the cortex was impacted using a metal pin (5 m s^−1^, 0.6 mm, 150 ms). The control group underwent craniotomy without CCI injury. Mice were monitored and returned to their cages after recovery.

### Immunofluorescence Staining

Mice were deeply anesthetized and perfused with ice‐cold 0.01 m PBS followed by 4% paraformaldehyde. The brains were fixed for 24 h, dehydrated with sucrose, and embedded in OCT. Ten‐micrometer coronal sections were cut and fixed in 4% paraformaldehyde, then rinsed with PBST and blocked with BSA. The sections were incubated with primary antibodies (GFAP, IBA‐1, Fluorojade B) overnight at 4 °C, washed, and treated with secondary antibodies for 2 h at room temperature. They were stained with DAPI for 15 s, rinsed with PBST, and imaged using fluorescence microscopy.

### Hematoxylin Staining

Mice from various treatment groups were euthanized after 28 days, dehydrated, and their hearts, livers, spleens, lungs, kidneys, and brains were imaged. Tissue sections, 10 µm thick, were prepared as previously described. The sections were fixed in 4% paraformaldehyde for 10 min, followed by a 2‐min rinse in distilled water. They were then incubated in hematoxylin solution (Beyotime) for 5 min and rinsed with distilled water for 10 min. Finally, the sections were rinsed twice with 70% alcohol and scanned.

### Nissl Staining

As previously described, the tissue sections were prepared and then dehydrated using a series of alcohol solutions: 75% alcohol for 15 min followed by 90% alcohol for 25 min. The sections were then rinsed with distilled water for 2 min. Subsequently, they were treated with Nissl staining solution (Beyotime) at room temperature for 5 min. After rinsing with distilled water, the sections were examined under a microscope.

### mNSS Score

The mNSS tests were performed on days −1, 1, 3, 7, 10, and 13 during behavioural experiments. The assessment covered motor function, sensory function, reflex responses, and balance. Scores ranged from 0 (normal) to 18 (maximum deficit).

### Wire Grip Test

The test was conducted on days −1, 1, 3, 7, 10, and 13 during behavioral experiments. Mice grasped a wire with their forelimbs, and the time they held on before falling was recorded to assess their motor function.

### Open Field Test

On the 15th day of behavioral experiments, the test was performed in a 40 × 40 cm arena. Before each trial, the arena was cleaned with 75% alcohol and air‐dried. Mice were placed at a fixed corner, and their movements were recorded. Center time and total distance traveled were analyzed using an automated tracking system.

### Morris Water Maze

The test was conducted from day 17 to 23 during behavioral experiments. A 1.2‐meter white pool was used, divided into four quadrants with consistent visual cues. A small platform was submerged 1–2 cm below the surface. Mice had to use learning and memory to locate the platform relative to the cues. Each trial began with mice facing the pool wall. On day 7, after six days of training, the platform was removed, and mice swam freely while their movements were recorded. Non‐swimming mice were excluded before the experiment. Behavioral tests were performed at 28 days to capture long‐term functional recovery, as cognitive deficits typically stabilize by 3–4 weeks post‐injury.

### Toxicity Assessment

On day 28 after the test, three mice from each group were euthanized. Serum was collected to assess liver function (AST, ALT, ALP) and renal function (CREA, BUN, UA). Major organs, including the brain, heart, spleen, liver, lungs, and kidneys, were excised for H&E staining.

Magnetic resonance imaging. T2‐weighted brain images were acquired using a 9.4 T small animal MRI scanner (Bruker BioSpec 94/30, USR). Mice were anesthetized with 1.5–2.0% isoflurane and scanned using a RARE sequence. Lesion volume and edema were assessed from hyperintense regions on T2‐weighted images using ImageJ. All analyses were performed in a blinded manner.

### Statistical Analysis

All quantitative data were presented as mean ± standard error of the mean (SEM), unless otherwise specified. Statistical analyses were performed using OriginPro 2022 (OriginLab, Northampton, MA, USA). For comparisons between two groups, an unpaired two‐tailed Student's *t*‐test was used. For comparisons among multiple groups, one‐way analysis of variance (ANOVA) followed by Tukey's post hoc test was applied. A value of *p* < 0.05 was considered statistically significant. Significance levels were denoted as follows: *p* < 0.05 (^*^), *p* < 0.01 (^**^), *p* < 0.001 (^***^). The exact n values (biological replicates for in vitro assays or animals per group for in vivo studies) are indicated in the figure legends. Each key experiment was independently replicated at least five times (in vitro) or included three or more animals per group (in vivo) to ensure reproducibility and robustness.

## Conflict of Interest

The authors declare no conflict of interest.

## Author Contributions

X.H., L.Z., and X.S. contributed equally to this work. Y.C., L.H., and L.D. conceived and designated the experiment. X.H., L.Z., and X.S. conducted the experiments, analyzed the data, wrote and edited the paper. C.M. and T.X. designed animal behavior experiments. M.C., X.S., W.F., and L.D. performed material development and analyzed the data. Y.C. and L.D. revised the final manuscript. Y.C. and L.H. supervised the study and commented on the project.

## Supporting information



Supporting Information

## Data Availability

The data that support the findings of this study are available from the corresponding author upon reasonable request.

## References

[1] N. F. Shaik , C. A. Law , H. Elser , A. L. C. Schneider , JAMA Neurol. 2023, 81, 194. 10.1001/jamaneurol.2023.4618 PMC10696510 38048122

[2] K. Blennow , D. L. Brody , P. M. Kochanek , H. Levin , A. McKee , G. M. Ribbers , K. Yaffe , H. Zetterberg , Nat. Rev. Dis. Primers 2016, 2, 16084. 27853132 10.1038/nrdp.2016.84

[3] K. Bowman , C. Matney , D. M. Berwick , JAMA, J. Am. Med. Assoc. 2022, 327, 419. 10.1001/jama.2022.0089 35103760

[4] W. Li , J. Qiu , X.‐L. Li , S. Aday , J. Zhang , G. Conley , J. Xu , J. Joseph , H. Lan , R. Langer , R. Mannix , J. M. Karp , N. Joshi , Sci. Adv. 2021, 7, abd6889. 10.1126/sciadv.abd6889 PMC7775748 33523853

[5] J. S. Y. Ahn , J. L. Gommerman , Nat. Rev. Immunol. 2025, 25, 234. 40032991 10.1038/s41577-025-01154-5

[6] B. Wang , Q. Zhang , C. Liu , X. Chen , Biomaterials 2025, 318, 123181. 39970603 10.1016/j.biomaterials.2025.123181

[7] H. Li , D. Sun , Z. Zhao , J. Fang , M. Li , C. Lv , W. Zhou , N. Li , Y. Guo , Z. Cao , K. Liu , X. Chen , J. Nanobiotechnol. 2024, 22, 477. 10.1186/s12951-024-02753-5 PMC11320991 39135044

[8] W. B. Hubbard , H. J. Vekaria , G. V. Velmurugan , O. J. Kalimon , P. Prajapati , E. Brown , J. G. Geisler , P. G. Sullivan , J. Neurotrauma 2023, 40, 2396. 37476976 10.1089/neu.2023.0102 PMC10653072

[9] S. Izzy , T. Yahya , O. Albastaki , H. Abou‐El‐Hassan , M. Aronchik , T. Cao , M. G. De Oliveira , K.‐J. Lu , T. G. Moreira , P. D. Silva , M. L. Boucher , L. C. Beauchamp , D. S. LeServe , W. N. Brandao , A. Carolina Durão , T. Lanser , F. Montini , J.‐H. Lee , J. D. Bernstock , M. Kaul , G. Pasquarelli‐do‐Nascimento , K. Chopra , R. Krishnan , R. Mannix , R. M. Rezende , F. J. Quintana , O. Butovsky , H. L. Weiner , Nat. Neurosci. 2025, 28, 499. 40016353 10.1038/s41593-025-01877-7 PMC11893472

[10] X. Qiu , S. Ping , M. Kyle , L. Chin , L.‐R. Zhao , Cells 2023, 12, 705. 36899841 10.3390/cells12050705 PMC10000780

[11] L. M. Wangler , J. P. Godbout , Trends Neurosci. 2023, 46, 926. 37723009 10.1016/j.tins.2023.08.008 PMC10592045

[12] L. K. Ferrarelli , Science 2017, 356, 37. 10.1126/science.356.6333.37-r 28385994

[13] I. R. A. R. Helmrich , E. Czeiter , K. Amrein , A. Büki , H. F. Lingsma , D. K. Menon , S. Mondello , E. W. Steyerberg , N. von Steinbüchel , K. K. W. Wang , L. Wilson , H. Xu , Z. Yang , D. van Klaveren , A. I. R. Maas , Lancet Neurol. 2022, 21, 792. 35963262 10.1016/S1474-4422(22)00218-6

[14] S. Friberg , C. Lindblad , F. A. Zeiler , H. Zetterberg , T. Granberg , P. Svenningsson , F. Piehl , E. P. Thelin , Nat. Rev. Neurol. 2024, 20, 671. 39363129 10.1038/s41582-024-01024-z

[15] Y. Wang , M. Pu , J. Yan , J. Zhang , H. Wei , L. Yu , X. Yan , Z. He , ACS Nano 2023, 17, 472. 36574627 10.1021/acsnano.2c08982

[16] M. R. Alkaslasi , E. Y. H. Lloyd , A. S. Gable , H. Silberberg , H. E. Yarur , V. S. Tsai , M. Sohn , G. Margolin , H. A. Tejeda , C. E. Le Pichon , Nat. Commun. 2025, 16, 1097. 39870620 10.1038/s41467-025-56292-0 PMC11772587

[17] Y. Zhang , G. Wei , W. Liu , T. Li , Y. Wang , M. Zhou , Y. Liu , X. Wang , H. Wei , Nat. Rev. Methods Primers 2024, 4, 36.

[18] Y. Feng , X. Luo , Z. Li , X. Fan , Y. Wang , R.‐R. He , M. Liu , Nat. Commun. 2023, 14, 5083. 37607944 10.1038/s41467-023-40794-w PMC10444825

[19] W. Gao , J. He , L. Chen , X. Meng , Y. Ma , L. Cheng , K. Tu , X. Gao , C. Liu , M. Zhang , K. Fan , D.‐W. Pang , X. Yan , Nat. Commun. 2023, 14, 160. 36631476 10.1038/s41467-023-35828-2 PMC9834297

[20] S. Koo , H. S. Sohn , T. H. Kim , S. Yang , S. Y. Jang , S. Ye , B. Choi , S. H. Kim , K. S. Park , H. M. Shin , O. K. Park , C. Kim , M. Kang , M. Soh , J. Yoo , D. Kim , N. Lee , B.‐S. Kim , Y. Jung , T. Hyeon , Nat. Nanotechnol. 2023, 18, 1502. 37884660 10.1038/s41565-023-01523-y

[21] B. Jia , Y. Yao , Y. Xu , Y. Zhao , J. Li , Y. Wu , X. Zeng , S. Hong , Q. Li , X. Liu , C. Luo , Chem. Eng. J. 2025, 515, 163592.

[22] Z. Gong , Z. Chen , D. Li , X. Lu , J. Wu , H. Sun , X. Wang , S. Liu , X. Xia , F. Lu , J. Jiang , C. Sun , H. Wang , F. Zeng , X. Ma , J. Nanobiotechnol. 2025, 23, 29. 10.1186/s12951-025-03098-3 PMC11748312 39833803

[23] Y. G. Kim , Y. Lee , N. Lee , M. Soh , D. Kim , T. Hyeon , Adv. Mater. 2023, 36, 2210819. 10.1002/adma.202210819 36793245

[24] Z. Yuan , L. Huang , Y. Liu , Y. Sun , G. Wang , X. Li , J. A. Lercher , Z. Zhang , Angew. Chem., Int. Ed. 2023, 63, 202317339. 10.1002/anie.202317339 38085966

[25] S.‐E. Hong , J. H. An , S.‐L. Yu , J. Kang , C. G. Park , H. Y. Lee , D. C. Lee , H.‐W. Park , W.‐M. Hwang , S.‐R. Yun , M. H. Park , K. R. Yoon , S.‐H. Yoon , J. Biomed. Nanotechnol. 2020, 16, 1144. 33308381 10.1166/jbn.2020.2948

[26] B. Choi , M. Soh , Y. Manandhar , D. Kim , S. I. Han , S. Baik , K. Shin , S. Koo , H. J. Kwon , G. Ko , J. Oh , H. Hwang , T. Hyeon , S. J. Lee , Nanoscale 2019, 11, 19437. 31475711 10.1039/c9nr02648g

[27] W. Wang , K. Li , W. Ma , Y. Li , F. Liu , Y. Kong , L. Wang , F. Yi , Y. Sang , G. Li , H. Liu , J. Qiu , Nat. Mater. 2025, 24, 1137. 40329084 10.1038/s41563-025-02214-w

[28] D. Wei , M. Zeng , B. Su , Y. Zhang , J. Ding , C. Wu , J. Sun , L. Zhou , H. Yin , H. Fan , Chem. Eng. J. 2024, 484, 149521.

[29] A. P. Carlson , D. Hänggi , R. L. Macdonald , C. W. Shuttleworth , Curr. Neuropharmacol. 2020, 18, 65. 31560289 10.2174/1570159X17666190927113021 PMC7327937

[30] R. Pathak , R. P. Dash , M. Misra , M. Nivsarkar , Acta Pharm. Sin. B 2014, 4, 151. 26579378 10.1016/j.apsb.2014.02.002 PMC4590727

[31] D. Tomassoni , A. Lanari , G. Silvestrelli , E. Traini , F. Amenta , Clin. Exp. Hypertens. 2008, 30, 744. 19021025 10.1080/10641960802580232

[32] X. Wang , Y. Yin , H. Zhou , B. Chi , L. Guan , P. Li , J. Li , Y. Wang , Exploration 2024, 5, 20240036. 40395758 10.1002/EXP.20240036 PMC12087414

[33] G. C. Terstappen , A. H. Meyer , R. D. Bell , W. Zhang , Nat. Rev. Drug Discovery 2021, 20, 362. 33649582 10.1038/s41573-021-00139-y

[34] C. Wang , Y. Xue , T. Markovic , H. Li , S. Wang , Y. Zhong , S. Du , Y. Zhang , X. Hou , Y. Yu , Z. Liu , M. Tian , D. D. Kang , L. Wang , K. Guo , D. Cao , J. Yan , B. Deng , D. W. McComb , R. E. Parsons , A. M. Minier‐Toribio , L. M. Holt , J. Pan , A. Hashemi , B. H. Kopell , A. W. Charney , E. J. Nestler , P. C. Peng , Y. Dong , Nat. Mater. 2025, 10.1038/s41563-024-02114-5 .

[35] W. M. Bement , A. B. Goryachev , A. L. Miller , G. Von Dassow , Nat. Rev. Mol. Cell Biol. 2024, 25, 290. 38172611 10.1038/s41580-023-00682-z PMC12706751

[36] S. Modi , G. López‐Doménech , E. F. Halff , C. Covill‐Cooke , D. Ivankovic , D. Melandri , I. L. Arancibia‐Cárcamo , J. J. Burden , A. R. Lowe , J. T. Kittler , Nat. Commun. 2019, 10, 4399. 31562315 10.1038/s41467-019-12382-4 PMC6764964

[37] J. Meza‐Resillas , F. O'Hara , S. Kaushik , M. J. Stobart , N. Ahmadpour , M. Kantroo , J. Del Rosario , M. C. Rodriguez , D. Koval , C. Glück , B. Weber , J. L. Stobart , Neurotherapeutics 2025, 10.1016/j.neurot.2025.e00614. PMC12491807 40404520

