# Correction to “Nanocatalytic Neuroprotection and Neurological Recovery Post‐Traumatic Brain Injury”

**DOI:** 10.1002/advs.202520834

**Published:** 2025-12-25

Xinjie Hong, Liang Zhao, Xianzheng Sang, Chao Ma, Meiqi Chang, Xinran Song, Wei Feng, Tao Xu, Li Ding^*^, Yu Chen^*^, and Lijun Hou^*^

https://doi.org/10.1002/advs.202505962

**Correction 1**

In Figure S9a, the image representing the “CZs+NMDP” group was incorrectly duplicated from the “NMDP” group. This has been rectified by replacing the image with the correct version based on reevaluation of the original experimental data.


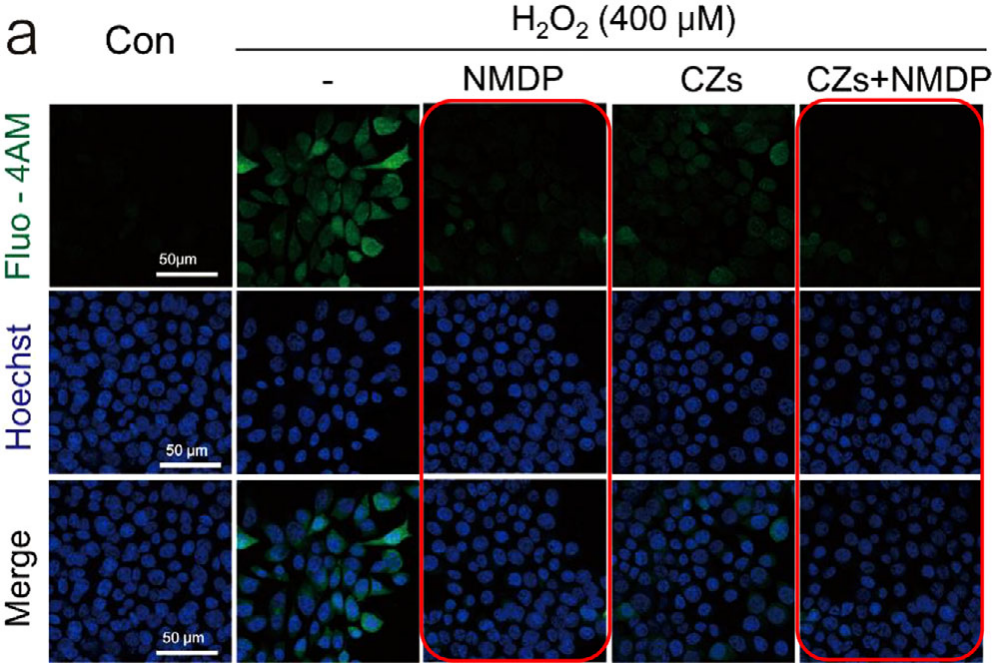


**Original Figure S9a**. Assessment of calcium homeostasis. Representative diagram of a confocal microscope. Scale bar: 50 µm.


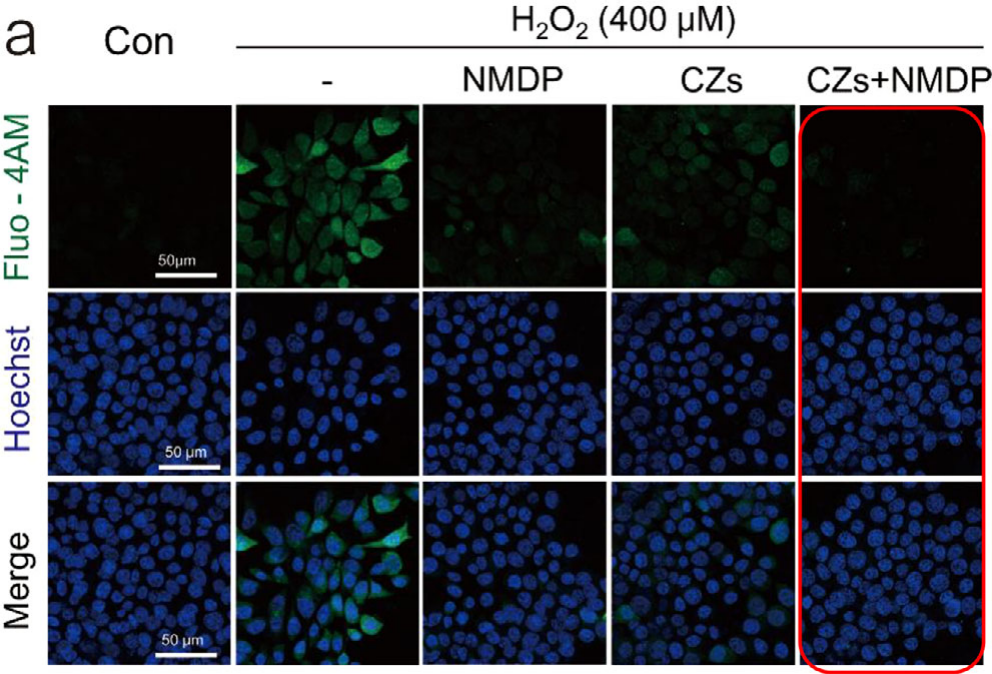


**Revised Figure S9a**. Assessment of calcium homeostasis. Representative diagram of a confocal microscope and. Scale bar: 50 µm.

**Correction 2**

In Figure S16, the scanning image for the TBI group was erroneously used due to a typesetting error. This has been corrected.


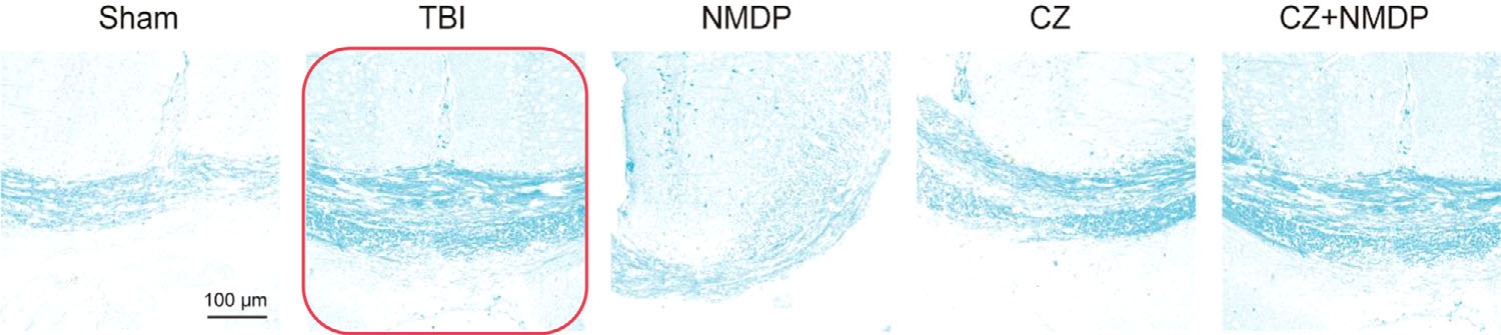


**Original Figure S16**. Representative pictures showing the results of LFB staining after various treatments. Scale bar: 100 µm.


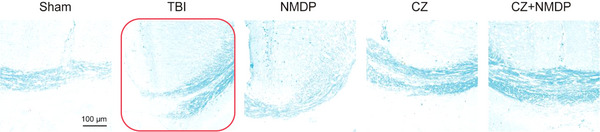


**Revised Figure S16**. Representative pictures showing the results of LFB staining after various treatments. Scale bar: 100 µm.

**Correction 3**

The panel labels (a–i) in Figure 6 were inadvertently omitted in the final published version. These labels have now been reinstated to accurately reflect the original data and ensure correct interpretation.


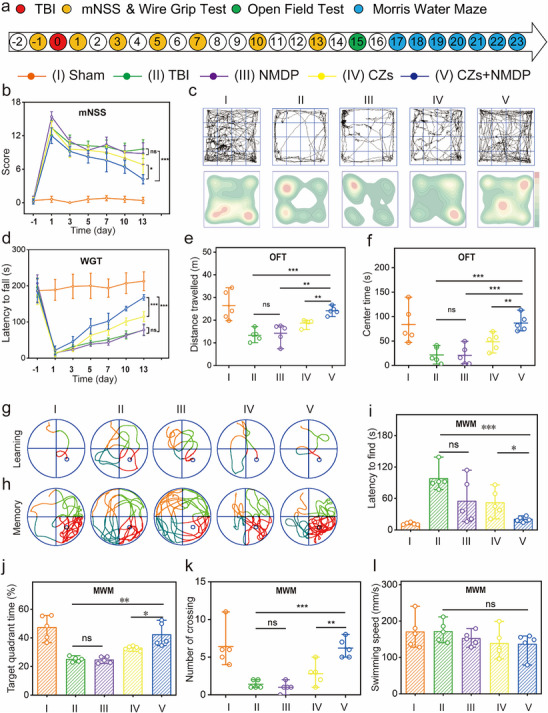


**Revised Figure 6**. Synergistic effects of CZs and nimodipine in improving long‐term neurologic function after TBI in mice (a) Time course diagram and experimental design of mouse experiments. (b) The mNSS scores were calculated after various treatments. (c) Images of open field test trajectories and heat maps for assessing locomotor behavior. (d) Motor function was evaluated by the hanging wire grip test. (e, f) Total distance and center time for each group in the open field test. (g, h) Representative images of the swimming trajectories during the learning phase and memory phase. The latency (i), target quadrant time (j), and crossing number (k) of the Morris water maze. (l) Swimming speed of mice in each group (n = 5). Statistical significance was analyzed using one‐way analysis of variance (ANOVA).

We sincerely apologize for these errors.

