# Supplementary material for: Nanocatalytic Neuroprotection and Neurological Recovery Post‐Traumatic Brain Injury
Source: Adv Sci (Weinh). 2025 Aug 11;12(41):e05962. doi: 10.1002/advs.202505962 (PMC12591159; doi:10.1002/advs.202505962)
Supplement: Supplementary file 1 — Supporting Information [file ADVS-12-e05962-s001.docx]

Supporting information for

Nanocatalytic Neuroprotection and Neurological Recovery Post-Traumatic Brain Injury

*Xinjie Hong,* *Liang Zhao, Xianzheng Sang, Chao Ma, Meiqi Chang, Xinran Song, Wei Feng, Tao Xu, Li Ding*, Yu Chen* and Lijun Hou**

X. Hong, L. Zhao, X. Sang, C. Ma, Prof. T. Xu, Prof. L. Hou

Department of Neurosurgery, The Second Affiliated Hospital of Naval Medical University, Shanghai 200003, P. R. China.

Email: houlijun@smmu.edu.cn.

Dr. M. Chang

Laboratory Center, Shanghai Municipal Hospital of Traditional Chinese Medicine, Shanghai University of Traditional Chinese Medicine, Shanghai 200071, P. R. China.

Prof. L. Ding

Department of Medical Ultrasound, Shanghai Tenth People's Hospital, Tongji University Cancer Center, School of Medicine, Tongji University, Shanghai 200072, P. R. China;

Email: dingli@tongji.edu.cn.

X. Song, Prof. W. Feng and Y. Chen

Materdicine Lab, School of Life Sciences, Shanghai University, Shanghai 200444, P. R. China.

Email: chenyuedu@shu.edu.cn.

Supporting Information

Supplementary Table

Table S1.Glossary of Abbreviations

| Abbreviation | Full Term |
| --- | --- |
| TBI | Traumatic brain injury |
| BBB | Blood–brain barrier |
| CZs | Cerium-zirconia nanozymes |
| CNS | Central nervous system |
| ROS | Reactive oxygen species |
| SOD | Superoxide dismutase |
| CAT | Catalase |
| POD | Peroxidase |
| HRTEM | High-resolution transmission electron microscopy |
| EDS | Energy dispersive X-ray spectroscopy |
| XRD | X-ray diffraction |
| FTIR | Fourier transform infrared spectroscopy |
| XPS | X-ray photoelectron spectroscopy |
| DLS | Dynamic light scattering |
| PEG | Polyethylene glycol |
| JC-1 | 5,5’,6,6’-Tetrachloro-1,1’,3,3’-tetraethylbenzimidazolylcarbocyanine iodide |
| SEM | Standard error of the mean |
| ANOVA | Analysis of variance |
| PBS | Phosphate-buffered saline |
| DMEM | Dulbecco’s Modified Eagle Medium |
| FJB | Fluoro-Jade B |
| IHC | Immunohistochemistry |
| Iba-1 | Ionized calcium-binding adapter molecule 1 |
| GFAP | Glial fibrillary acidic protein |
| CaMKII | Calcium/calmodulin-dependent protein kinase II |
| H&E | Hematoxylin and eosin |
| MRI | Magnetic resonance imaging |
| mNSS | Modified neurological severity score |
| AST | Aspartate transaminase |
| ALT | Alanine transaminase |
| ALP | Alkaline phosphatase |
| CREA | Creatinine |
| BUN | Blood urea nitrogen |
| UA | Uric acid |
| RNA-seq | RNA sequencing |
| DEG | Differentially expressed genes |
| GO | Gene Ontology |
| KEGG | Kyoto Encyclopedia of Genes and Genomes |
| MWM | Morris water maze |
| OFT | Open field test |
| WGT | Wire grip test |

Supplementary Figures


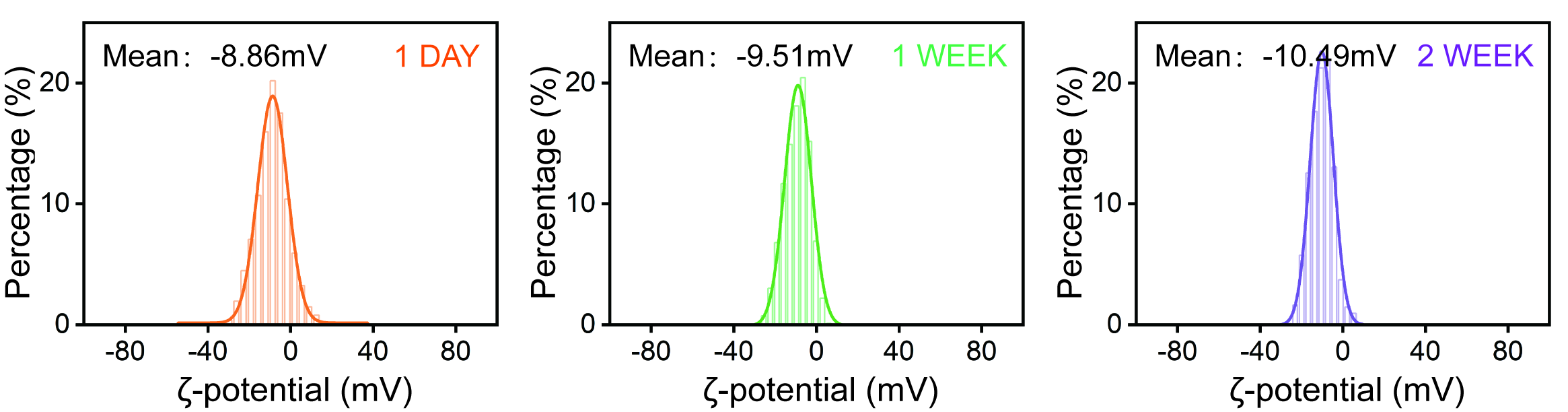


Figure S1. Zeta potential measurements at multiple time points.


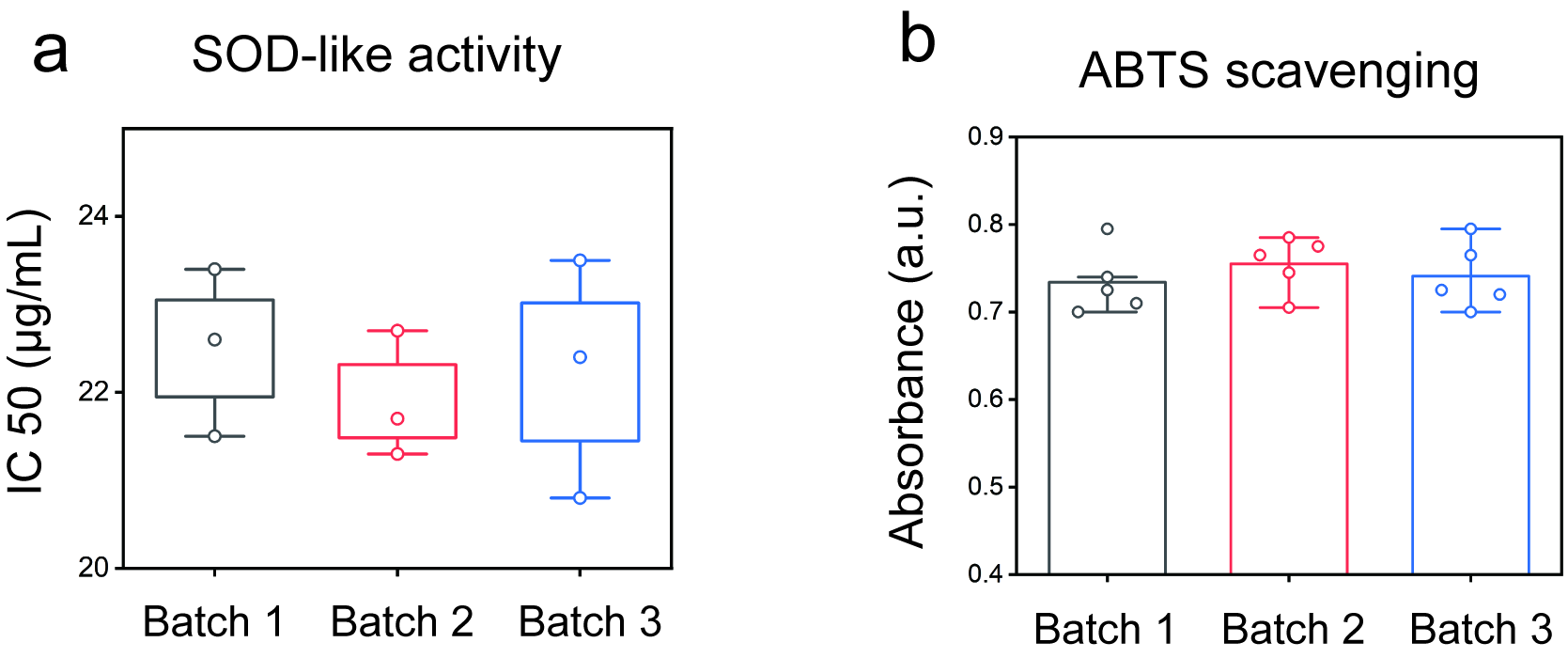


Figure S2. (a) IC50 values of SOD-mimetic activity across three independent CZ synthesis batches (P = 0.1123). (b) ABTS scavenging of CZs across three independent batches at a concentration of 60 μg/mL (P = 0.651).


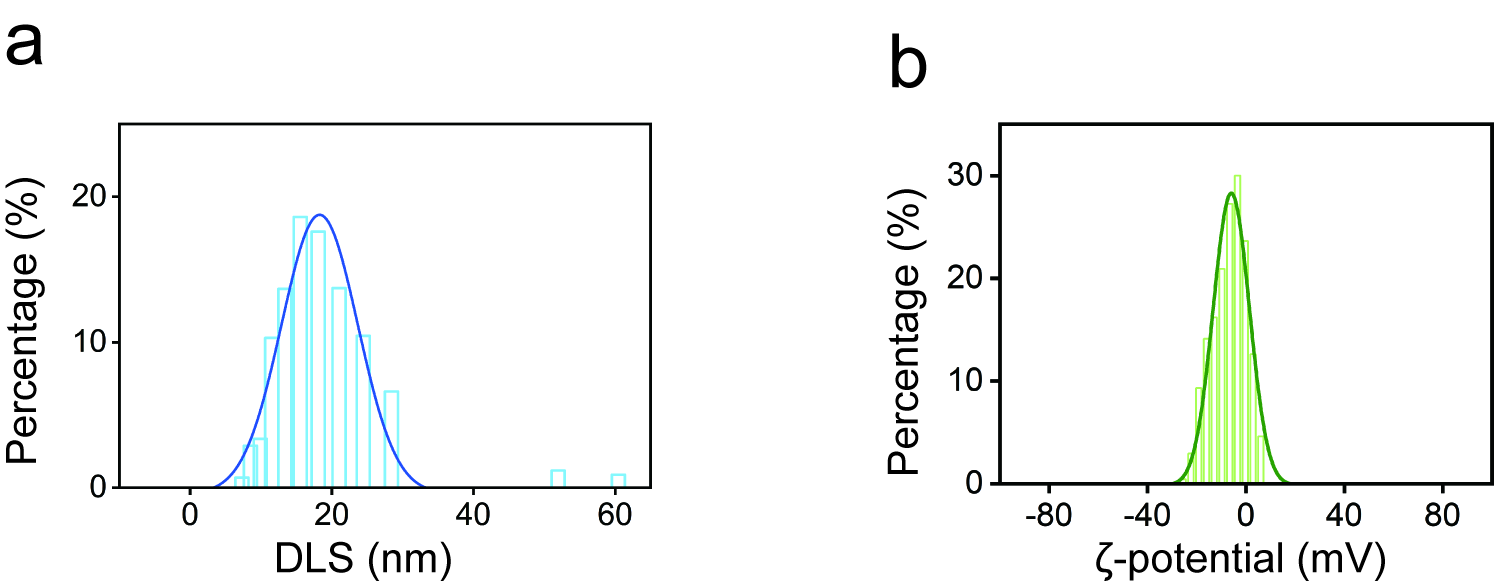


Figure S3. (a) Hydrodynamic diameter of CZs after 24-hour incubation at 37 °C in human plasma reference material. (b) Zeta potential of CZs measured after plasma incubation.


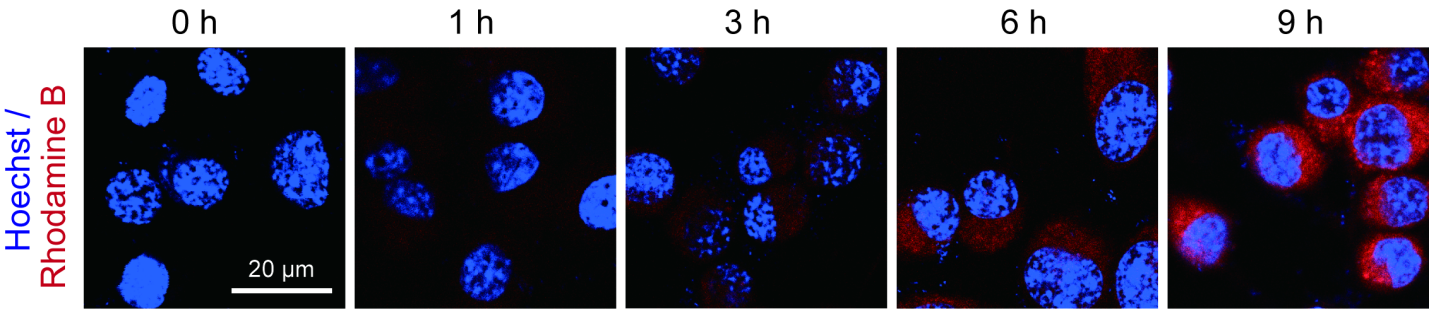


Figure S4. Progressive internalization of nanozymes. Increasing red fluorescence in rhodamine B-labeled CZs. Scale bar: 20 μm.


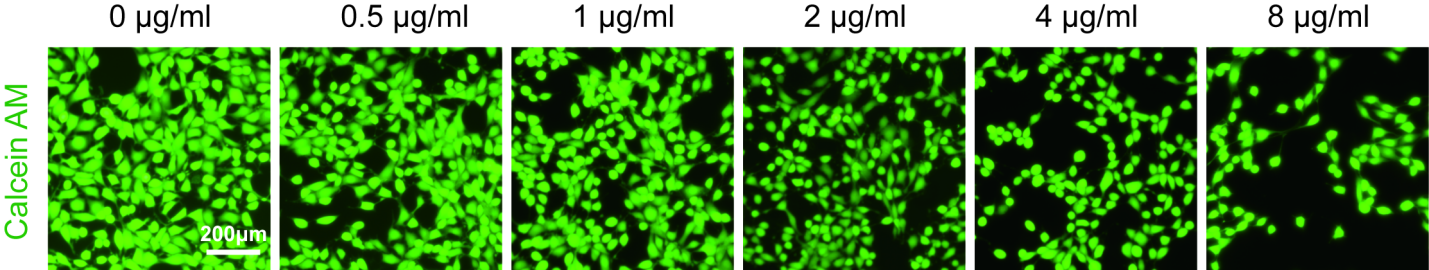


Figure S5. Cytotoxicity assessment of nanozymes. Comprehensive evaluation of nanozyme cytotoxicity using cytofluorescence analysis. Scale bar: 200 μm.


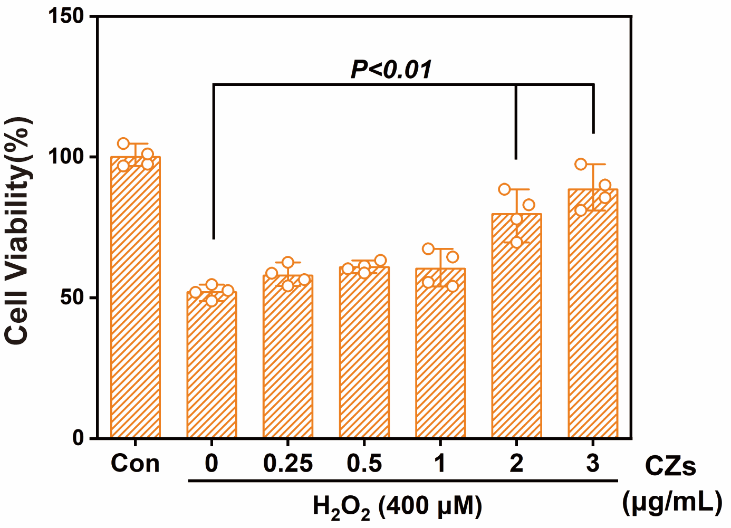


Figure S6. Effects of CZs in HT22 cells oxidative stress. Results of CZs application alone.


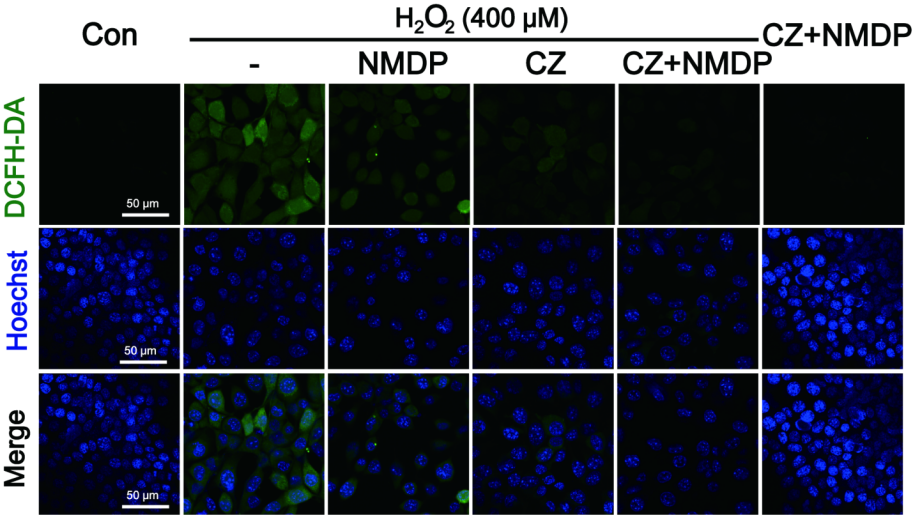


Figure S7. Fluorescence microscopy of HT22 cells stained with DCFH-DA. Scale bar: 50 μm.


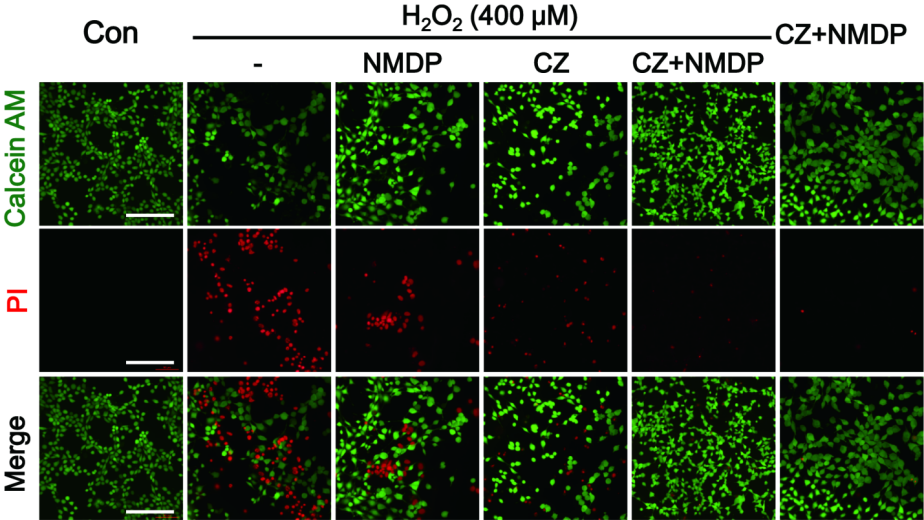


Figure S8. Qualitative assessment of cell therapy. Results were evaluated using calcein AM/PI assay, with green/red fluorescence visualized via fluorescence microscopy. Scale bar: 200 μm.


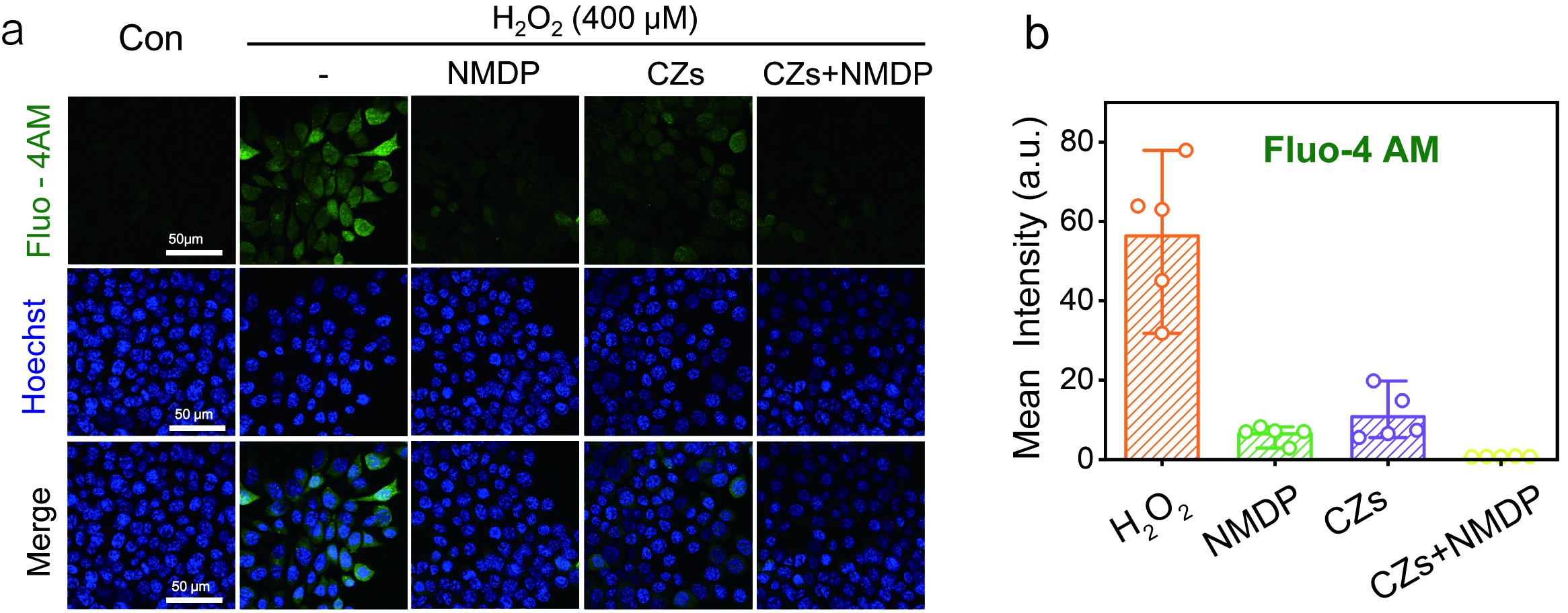


Figure S9. (a) Assessment of calcium homeostasis. Representative diagram of a confocal microscope and (b) quantification. Scale bar: 50 μm.


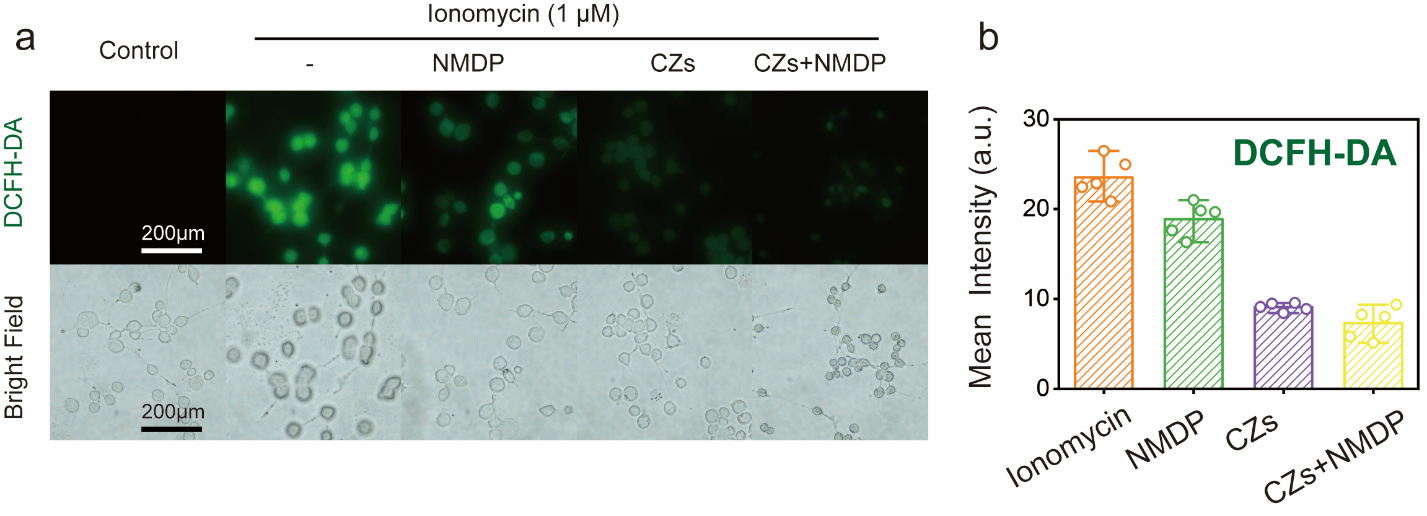


Figure S10. (a) Representative DCFH-DA fluorescence images of HT22 cells. (b) Quantification of ROS levels based on DCFH-DA fluorescence intensity(n=5). Scale bar: 200 μm.


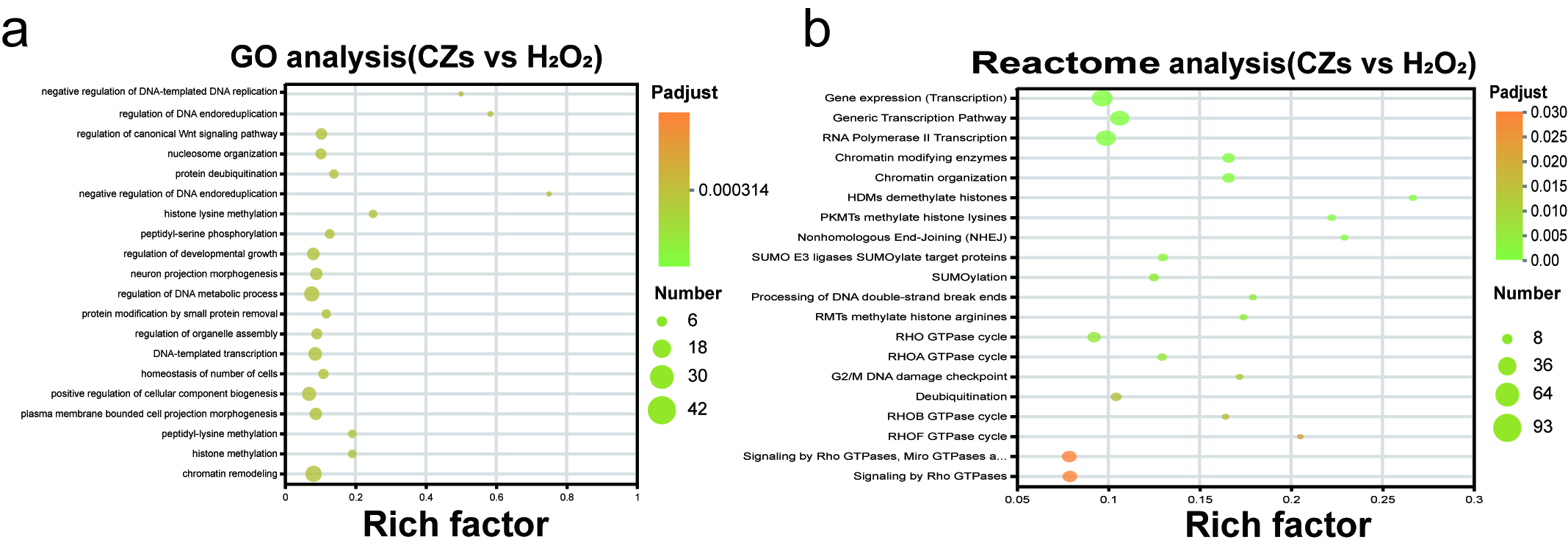


Figure S11. (a) GO clustering analysis. (b) Reactome clustering analysis.


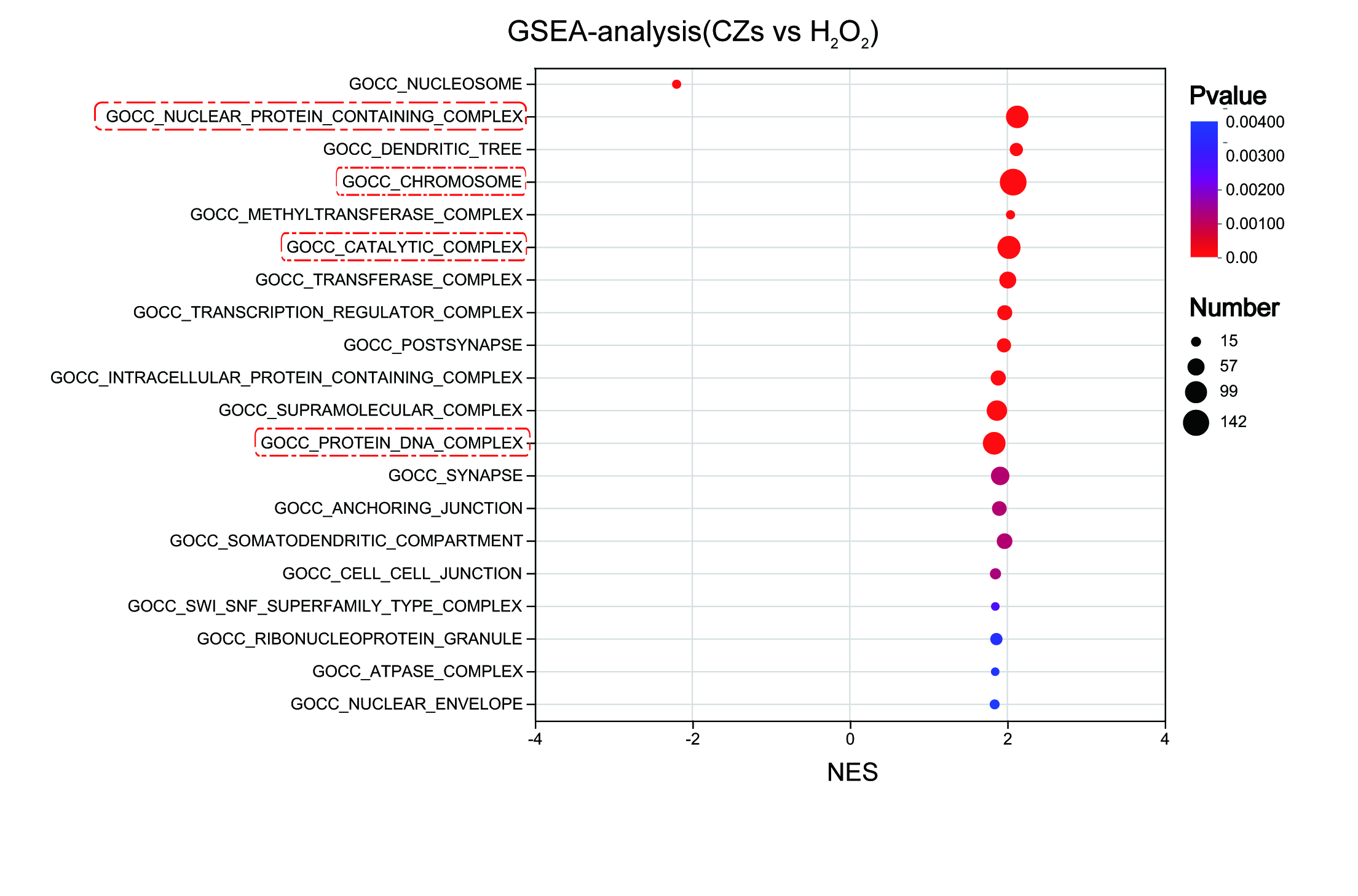


Figure S12. Gene set enrichment analysis (GSEA) to analyze DEG between the CZs group and the H_2_O_2_ group.


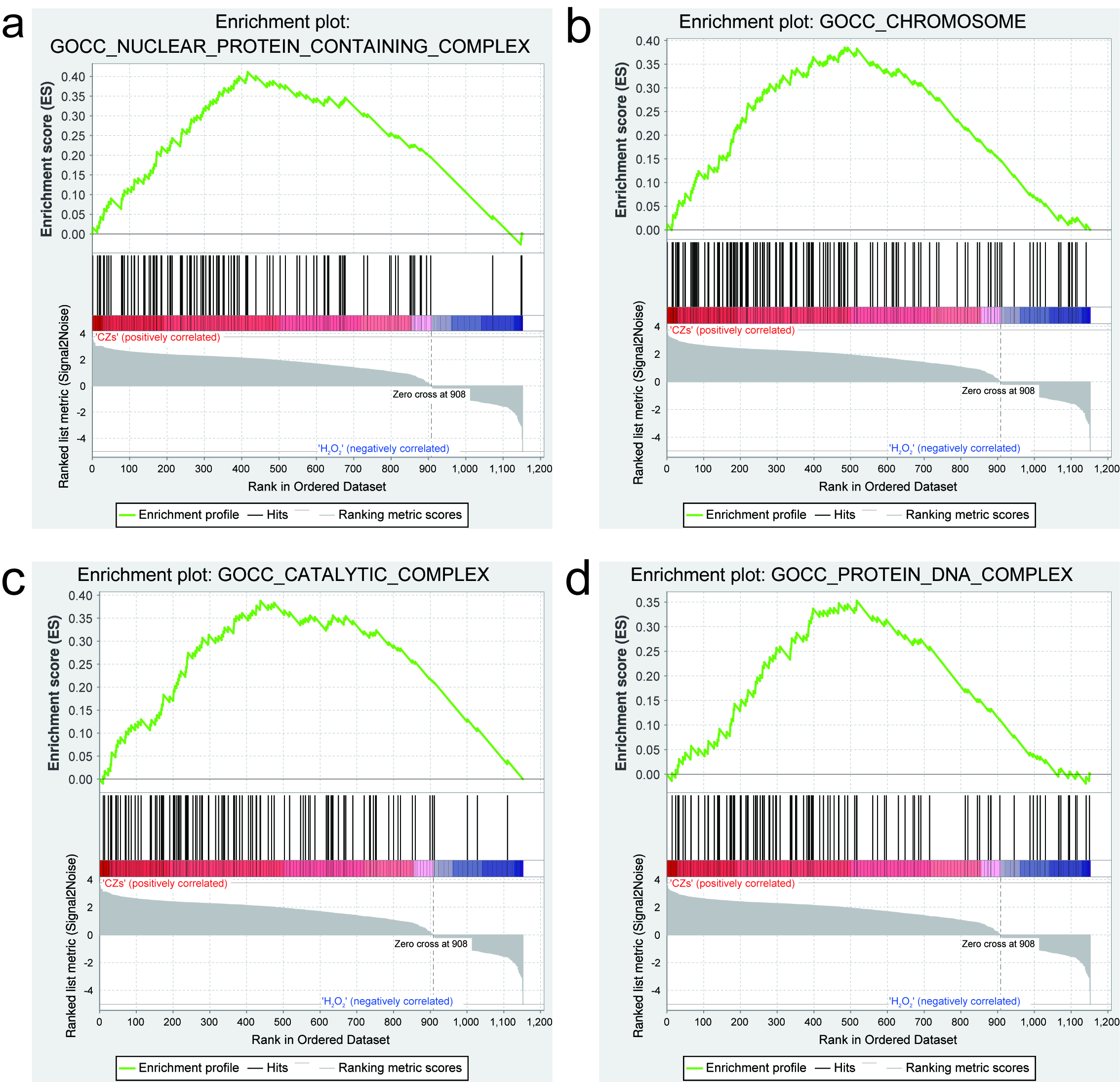


Figure S13. GSEA enrichment plots for selected gene sets. (a) Enrichment plot for genes related to chromosomal structure. (b)Enrichment plot for genes encoding nuclear proteins. (c) Enrichment plot for genes involved in catalytic activities. (d)Enrichment plot for genes encoding DNA-interacting proteins. Positive ES values indicate upregulated genes, while negative values indicate downregulated genes.


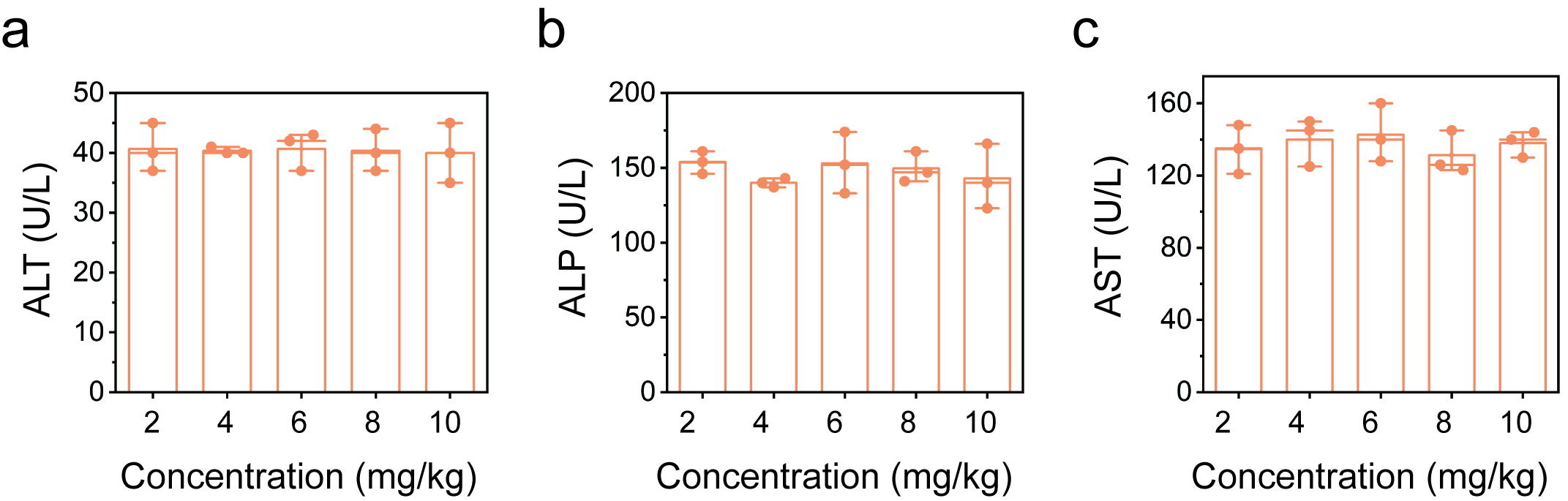


Figure S14. The ALT(a), ASP (b), AST (c) after injection of CZs at doses of 2, 4, 6, 8, and 10 mg/kg within one week.


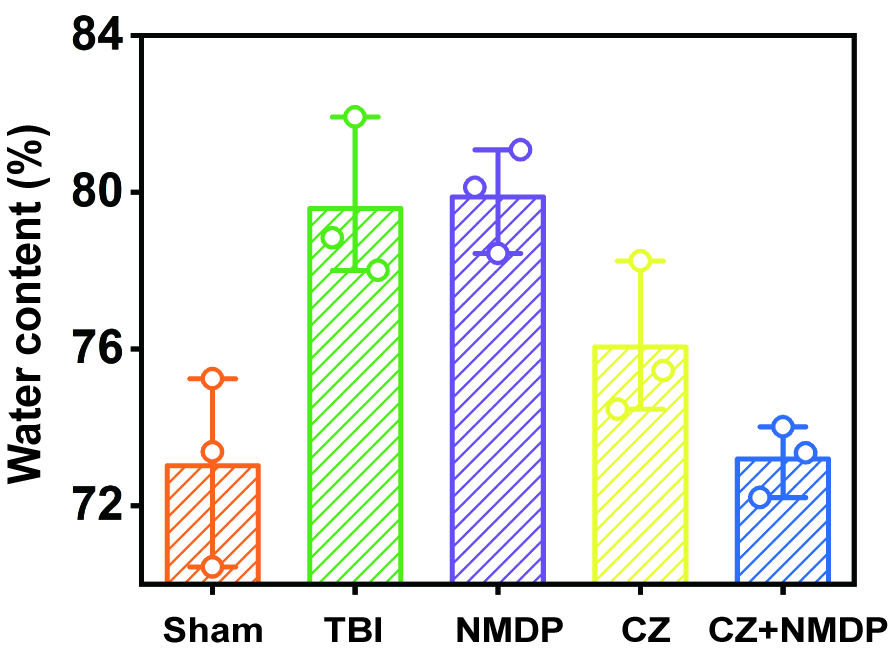


Figure S15. Water content in brain tissue of each group (n = 3).


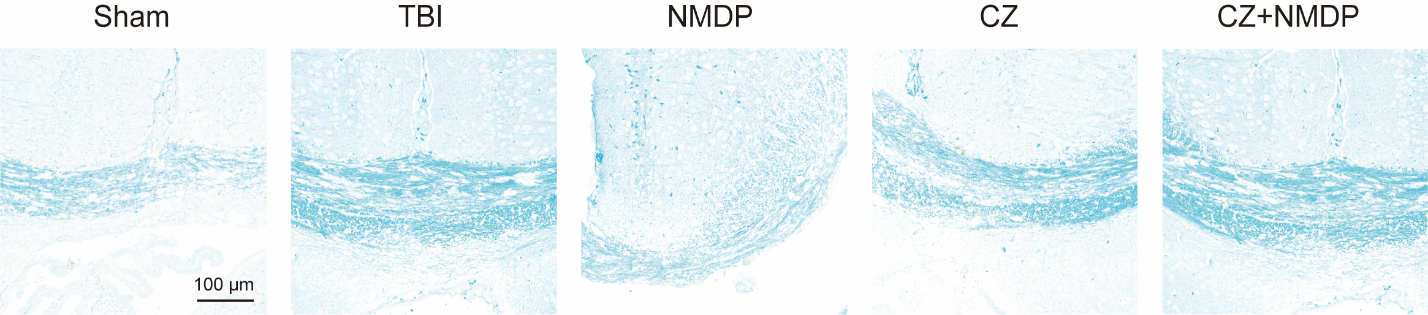


Figure S16. Representative pictures showing the results of LFB staining after various treatments. Scale bar: 100 μm.


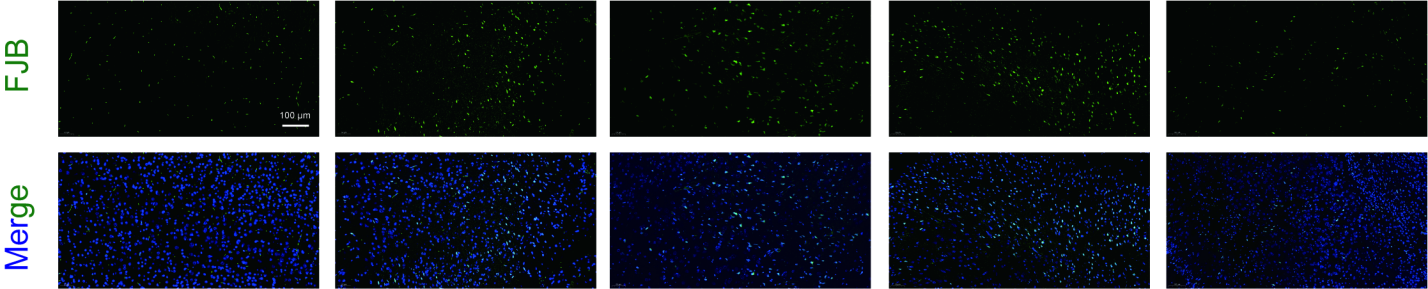


Figure S17. Representative pictures showing the results of FJB staining after various treatments. Scale bar: 100 μm.


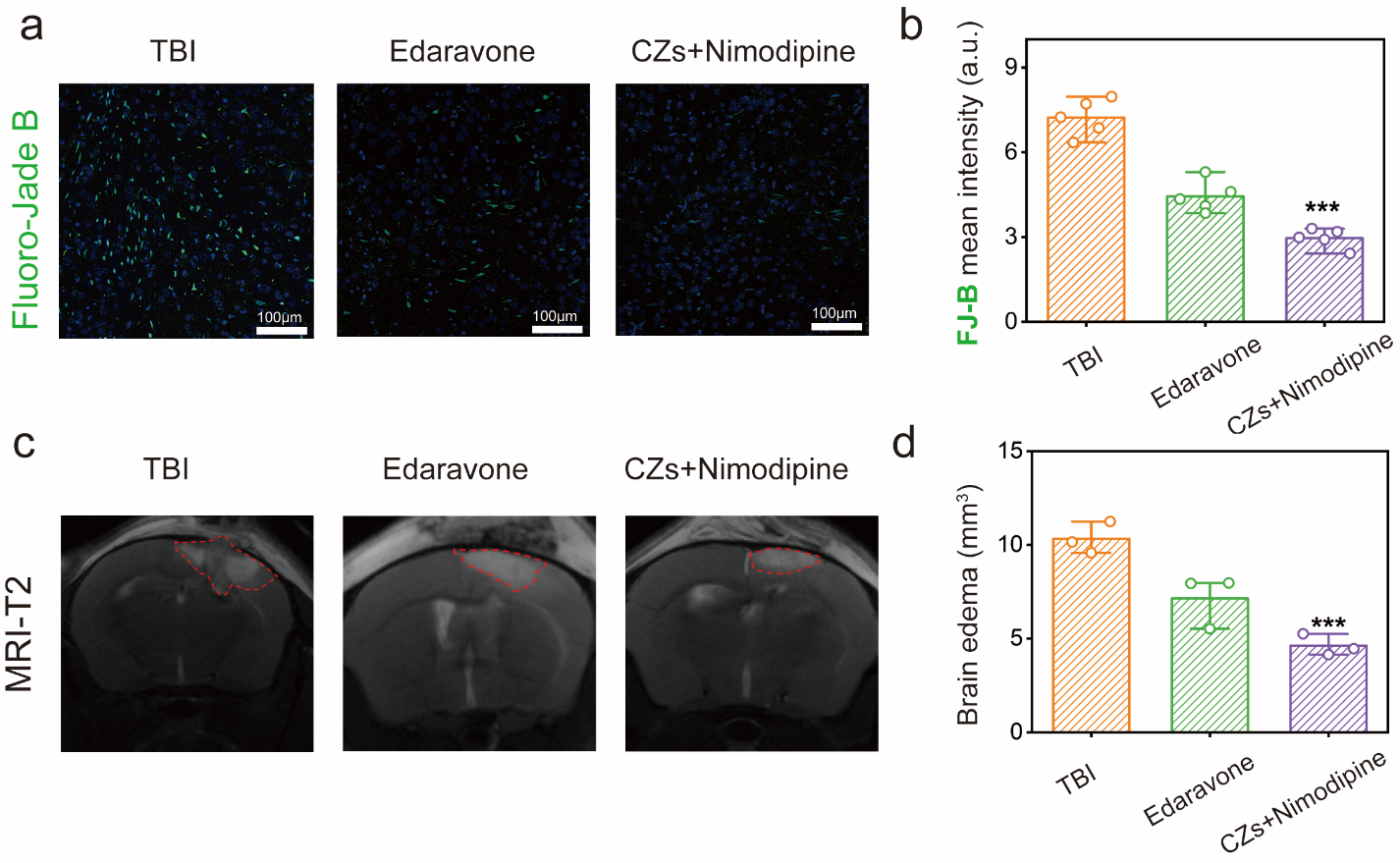


Figure S18.Comparative evaluation of neuroprotective efficacy between the CZs + nimodipine combination and edaravone monotherapy. (a) Representative images of Fluoro-Jade B (FJB) staining in brain sections from shame, edaravone-treated, and CZs + nimodipine–treated groups at day 7 post-injury. (b)Quantification of FJB-positive cells in the cortex (n = 5/group). (c) MRI T2-weighted images showing brain edema in each group at day 7 post-injury. (d) Quantification of edema volume based on hyperintense regions in T2-weighted MRI images (n = 3/group).


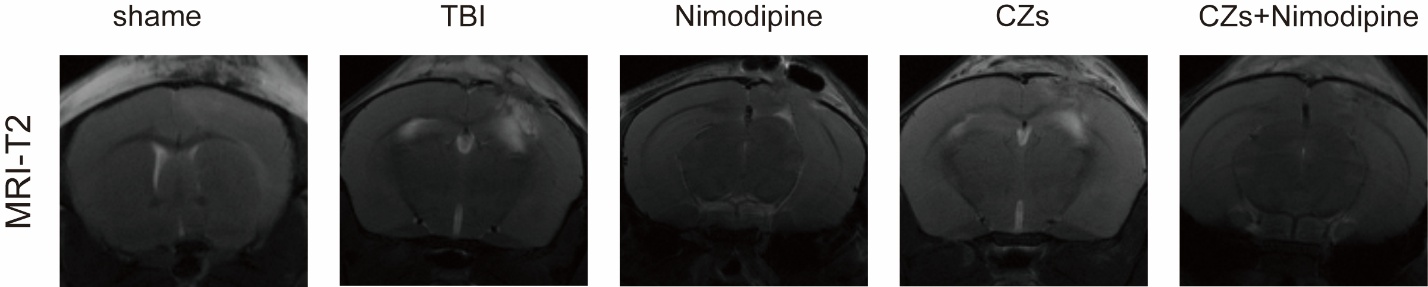


Figure S19.Representative pictures showing the lesion volume based on MRI-T2.


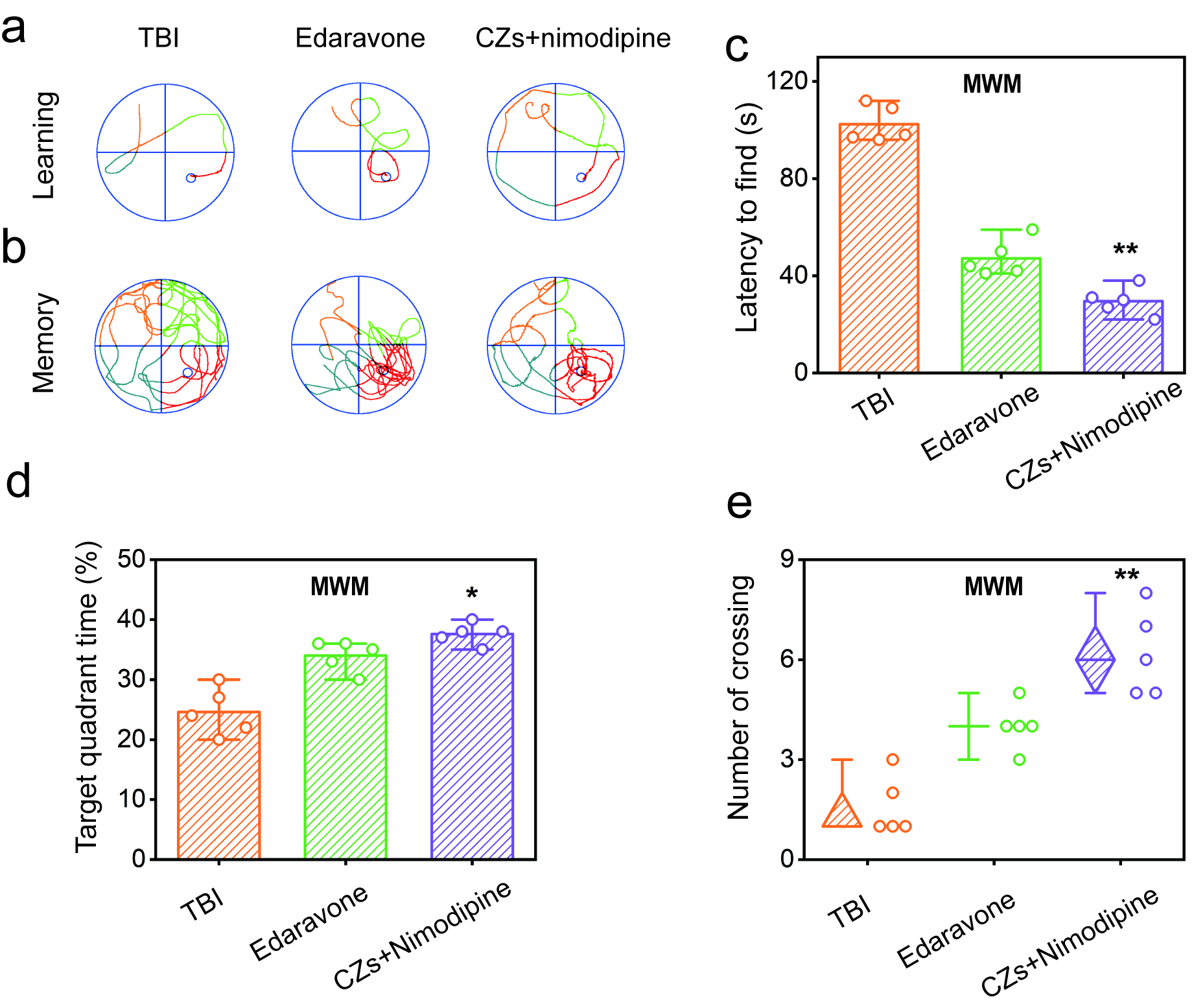


Figure S20. (a, b) Representative images of the swimming trajectories during the learning phase and memory phase. The latency (c), target quadrant time (d) and crossing number (e) of the Morris water maze. Statistical significance was analyzed using one-way analysis of variance (ANOVA). The levels of significance were set at P < 0.05 (*), P < 0.01 (**), and P < 0.001 (***).


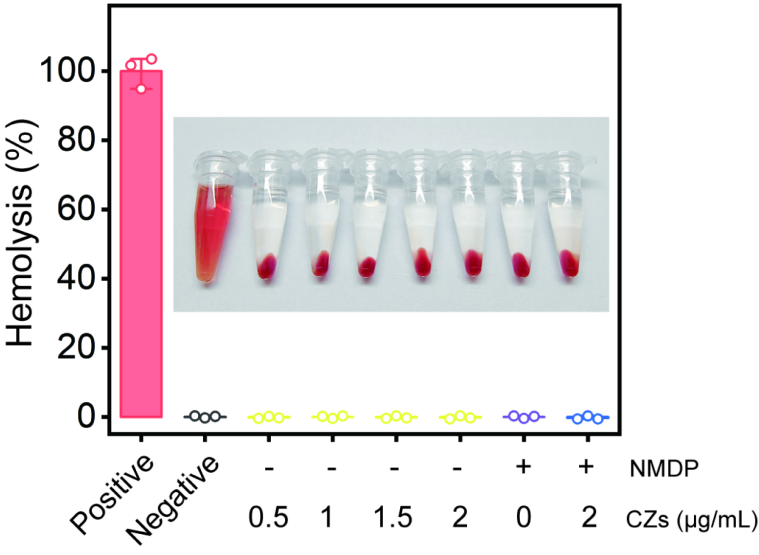


Figure S21. *In vitro* hemolysis assay for CZ nanozymes.


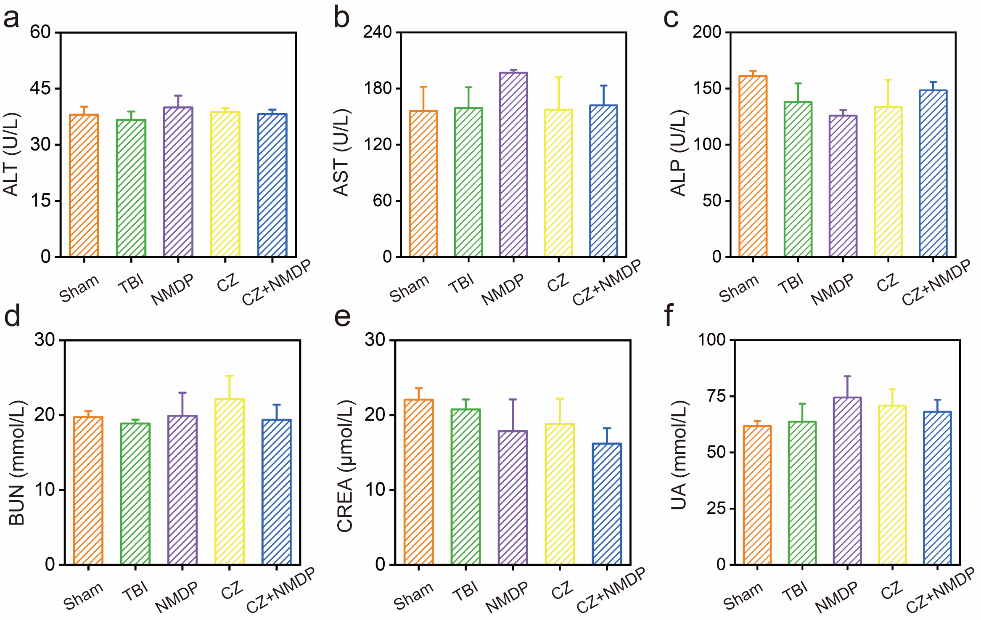


Figure S22. The ALT(a), AST (b), ALP (c), BUN (d), serum CREA (e) and UA(f) after CZs i.p. injection (n=5).


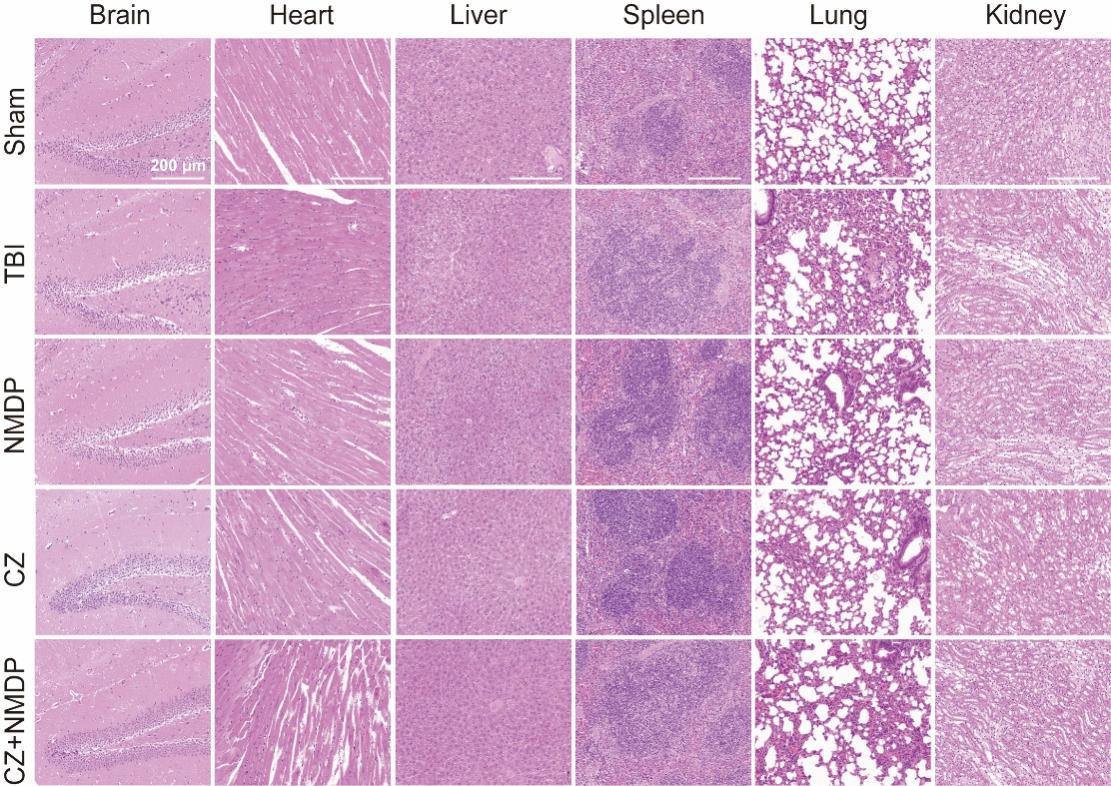


Figure S23. H&E staining of brain, heart, liver, spleen, lung and kidney after treatment after various group. Scale bar: 200 μm.


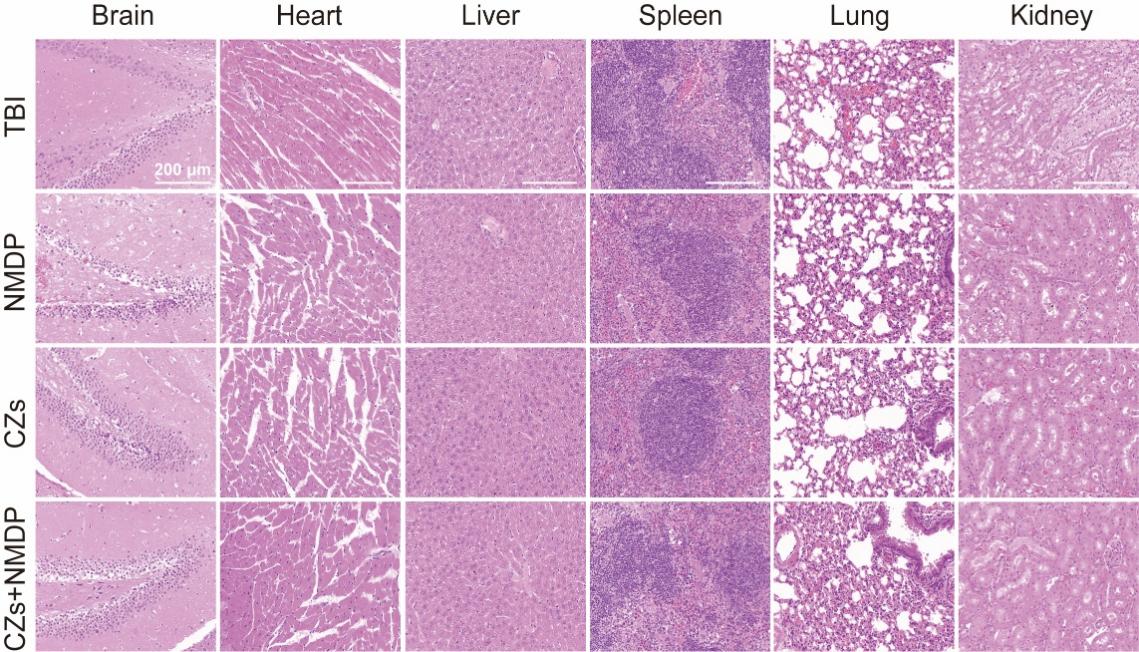


Figure S24. H&E staining of major organs at 42 days post-treatment to assess long-term biosafety of CZs and combination therapy.Scale bar: 200 μm.
